# Local Mo^6+^ Coordination and Textural Accessibility, Rather than Bulk Phase, Correlate with the Peroxidase-Mimetic Reactivity of CoMoO_4_ Nanopowders

**DOI:** 10.3390/ma19204318

**Published:** 2026-10-14

**Authors:** Milena Rosić, Zorica Mojović, Maja C. Pagnacco, Jelena V. Senćanski, Jelena P. Maksimović, Dragana Jordanov, Maria Čebela

**Affiliations:** 1 “Vinča” Institute of Nuclear Sciences, National Institute of the Republic of Serbia, University of Belgrade, Mike Petrovića Alasa 12-14, 11351 Belgrade, Serbia; mrosic@vin.bg.ac.rs (M.R.); djordano@vin.bg.ac.rs (D.J.); 2 Institute of Chemistry, Technology and Metallurgy, University of Belgrade, Njegoševa 12, 11000 Belgrade, Serbia; zorica.mojovic@ihtm.bg.ac.rs (Z.M.); maja.pagnacco@ihtm.bg.ac.rs (M.C.P.); 3 Institute for Multidisciplinary Research, University of Belgrade, Kneza Višeslava 1, 11030 Belgrade, Serbia; jelenasencanski@gmail.com; 4 Faculty of Physical Chemistry, University of Belgrade, Studentski trg 12-16, 11158 Belgrade, Serbia; jelena.maksimovic@ffh.bg.ac.rs

**Keywords:** CoMoO_4_ nanopowders, glycine nitrate process, FTIR coordination analysis, local coordination heterogeneity, Briggs–Rauscher reaction, Lewis acidity, peroxidase mimic, calcination, electrochemical impedance spectroscopy

## Abstract

**Highlights:**

- ATR-FTIR indicates octahedral-like (α-type) Mo^6+^ environments in CoMoO_4_ powders classified as *β*-phase by XRD.
- The nominally *β*-CoMoO_4_ powder calcined at 450 °C is the most active sample in the Briggs–Rauscher and voltammetric tests.
- Briggs–Rauscher oscillations discriminate the H_2_O_2_ reactivity of the three powders with high mass sensitivity.
- The descriptor *Φ* = *ρ_α_* × *S_BET_* identifies the most active powder; its limitations are discussed.
- Bulk phase identity alone does not predict peroxidase-mimetic activity in this CoMoO_4_ series.

**Abstract:**

Whether the bulk crystallographic phase determines the H_2_O_2_ reactivity of oxide nanopowders has direct implications for catalyst design. Three glycine–nitrate-derived CoMoO_4_ nanopowders—as-synthesized, calcined at 450 °C (both *β*-CoMoO_4_ by XRD), and calcined at 1000 °C (*α*-CoMoO_4_)—were studied by ATR-FTIR spectroscopy, the Briggs–Rauscher (BR) oscillatory reaction, cyclic voltammetry (CV), and electrochemical impedance spectroscopy. Deconvolution of the Mo–O stretching region with a uniform Voigt line shape resolves octahedral (*α*-type) and tetrahedral (*β*-type) Mo^6+^ components in both β-classified powders, indicating short-range coordination heterogeneity not detected by XRD. In the BR reaction (n = 3), the 450 °C powder suppresses oscillations at ≤0.002 g, whereas the as-synthesized powder still oscillates at 0.040 g (slope of the τ_osc_ decrease is 5172 ± 406 s g^−1^), and the bulk-*α* 1000 °C powder is about four times less active per gram than the as-synthesized powder. CV supports the highest activity of the 450 °C powder. A semi-quantitative descriptor, *Φ* = *ρ_α_* × *S_BET_*, combining the FTIR *α*-type band-area fraction with the BET surface area, identifies the most active powder but does not discriminate between the two less active ones. Within this series, bulk phase identity alone does not predict peroxidase-mimetic activity.

## 1. Introduction

Cobalt molybdate (CoMoO_4_) has attracted sustained interest across a broad range of technological domains: as a supercapacitor electrode material [1,2,3], a heterogeneous catalyst [4,5], a photocatalyst [6], and a microwave dielectric ceramic [7]. Across applications, the surface chemistry of CoMoO_4_, rather than its bulk stoichiometry alone, is a key determinant of its functional performance. CoMoO_4_ is particularly versatile among AMoO_4_ compounds owing to the coexistence of two complementary properties: the Co^2+^/Co^3+^ faradaic redox couple and the Lewis acidity of Mo^6+^ centres. In the peroxidase-like decomposition of H_2_O_2_, Co^2+^/Co^3+^ redox cycling is thought to carry the electron-transfer step, while Lewis-acidic Mo^6+^ centres may facilitate substrate coordination and peroxide activation [8]. Further contributions can arise from oxygen vacancies, which act as adsorption and activation sites for H_2_O_2_ and modify the electronic structure of neighbouring metal centres; from surface hydroxylation, which provides Brønsted sites and affects the adsorption of H_2_O_2_; and from defects, including undercoordinated Co and Mo at grain boundaries and in poorly crystalline domains [8]. Through these contributions, CoMoO_4_ behaves as a peroxidase mimic, catalyzing the decomposition of H_2_O_2_ in a manner that has been compared with the iron-porphyrin active sites of natural peroxidase enzymes, in which the peroxide is activated through coordination to a Lewis-acidic metal centre [8]. Lewis acid sites on transition-metal oxide surfaces are coordinatively unsaturated metal centres capable of accepting electron pairs from adsorbed nucleophiles [9,10]. On the basis of coordination-chemistry arguments and the prior literature, Mo^6+^ in octahedral-like coordination, which exposes short, high-bond-order terminal Mo–O bonds, is expected to be more Lewis-acidic and to activate oxidative substrates more readily than Mo^6+^ in the fully saturated MoO_4_ tetrahedra of the *β*-polymorph [8,11]. This expectation was not tested directly in the present study.

CoMoO_4_ exists in two monoclinic polymorphs that differ fundamentally in their Mo^6+^ local coordination geometry. In the *β*-phase (*C2*/*m*, ICSD #78328), Mo^6+^ occupies isolated tetrahedral MoO_4_ sites (Mo–O ≈ 1.82 Å). In the *α*-polymorph (*C2*/*m*, ICSD #23808), both Co^2+^ and Mo^6+^ are octahedrally coordinated: Mo^6+^ occupies edge-sharing MoO_6_ octahedra assembled into Mo_4_O_16_ tetramers, with Mo–O bond lengths spanning 1.73–2.25 Å, and the unit-cell volume is smaller (≈601 vs. ≈703 Å^3^) [11,12,13]. Which polymorph is observed at room temperature depends on thermal history, mechanical stress and crystallite size: the *β*-phase can be retained metastably, particularly in nanocrystalline powders, whereas grinding or pressure favours the α-form [9,14]. For the GNP-derived powders studied here, complete conversion to *α*-CoMoO_4_ was observed only after calcination at 1000 °C, above the DTA event at ≈913 °C [6].

Shifting from tetrahedral to octahedral Mo^6+^ coordination may therefore have surface-chemical consequences. In *α*-CoMoO_4_, the distorted MoO_6_ octahedra expose short terminal Mo–O bonds (≈1.73 Å) at coordinatively unsaturated Mo^6+^ centres. On structural grounds, it is anticipated that these undersaturated sites carry higher Lewis acidity than the fully saturated MoO_4_ tetrahedra of *β*-CoMoO_4_ [11]; as a consequence, they have been proposed to activate H_2_O_2_ more effectively [8]. Kinetic measurements on cobalt–molybdenum oxide surfaces have been reported to be consistent with Lewis acid site density at Mo^6+^ playing a primary role in controlling oxidative surface reaction rates, with octahedral and tetrahedral coordination environments yielding measurably different activation parameters [15]. Mo^6+^ coordination geometry, through its expected effect on Lewis acidity, may therefore be regarded as one structural predictor of intrinsic per-site activity (as inferred from coordination-chemistry arguments and the literature cited rather than from direct measurement in the present study). The macroscopic rate per gram, however, is additionally governed by geometric accessibility and may be modulated by Co^2+^/Co^3+^ redox chemistry, oxygen vacancies and defect density [8]. On this basis alone, the bulk-α 1000 °C powder would be expected to be the most active peroxidase mimic in this series; the data presented below show that this expectation is not fulfilled.

The starting point for the present study is an earlier characterization of the same three CoMoO_4_ powders prepared by the glycine–nitrate process (GNP) [6], which established bulk XRD phase identities, FESEM morphology, BET textural parameters, and photocatalytic activity towards crystal violet under UV irradiation. The 1000 °C powder proved to be the most efficient photocatalyst, which was attributed to the ability of its well-crystallized, single-phase α-CoMoO_4_ structure to suppress bulk charge-carrier recombination rather than to its band gap [6]. Two questions lay outside the scope of that study: (1) whether the nominally single-phase *β* powders carry any local (short-range) coordination heterogeneity not detected by powder XRD; and (2) how the three powders rank when the performance criterion shifts from photocatalysis to reactivity towards H_2_O_2_.

In relation to the first question, there are good mechanistic reasons to expect coordination heterogeneity in GNP-derived products. First, a computational exploration of the CoMoO_4_ energy landscape has identified eleven structurally distinct polymorphic modifications within a narrow energy window of ≈20 kJ mol^−1^, all kinetically accessible at the temperatures reached during low-temperature synthesis [16]. The spatially disordered combustion front inherent to the GNP process can simultaneously nucleate and arrest multiple distinct local coordination environments in adjacent sub-micrometre domains, generating local Mo^6+^ coordination that diverges from what long-range powder XRD reports as a single phase. Similar behaviour has been reported for GNP-derived Co_0.9_R_0.1_MoO_4_ (R = Ho, Yb, Gd) across all calcination temperatures studied [17], suggesting that coordination heterogeneity may be a recurring characteristic of this synthesis route rather than a composition-specific anomaly. To the best of the authors’ knowledge, whether the bulk phase label reliably predicts peroxidase-mimetic reactivity in GNP-derived CoMoO_4_ and whether local coordination heterogeneity accessible by FTIR correlates with differences in reactivity have yet to be addressed for any binary cobalt–molybdenum oxide system.

To probe the reactivity of the powders towards H_2_O_2_ with high sensitivity, we use the Briggs–Rauscher (BR) oscillatory reaction [18,19,20]. In the BR system, H_2_O_2_ acts mainly as the reductant that converts iodate and hypoiodous acid to iodide, with O_2_ evolution, and regenerates Mn^2+^ from Mn^3+^ in the radical, autocatalytic HIO_2_/IO_2_• pathway [18,20]. Any insoluble surface that heterogeneously decomposes H_2_O_2_ therefore withdraws a key reactant from the oscillator and shortens the oscillatory time *τ_osc_*. The non-linearity of the autocatalytic feedback functions as a chemical amplifier: minor differences in the rate of H_2_O_2_ consumption are translated into qualitatively distinct macroscopic outcomes (e.g., shortened reaction time, distorted waveform, or complete oscillation suppression), which makes the BR reaction a highly sensitive kinetic discriminator; the same sensitivity underlies its established use for measuring the activity of antioxidants that scavenge its radical intermediates [21]. It should be noted, however, that dissolved transition-metal species released from the solid surface, including Co^2+^ ions and Mo-oxo complexes, may also interact with the autocatalytic Mn^2+^/Mn^3+^ redox couple and with iodine intermediates, thereby perturbing the oscillatory dynamics through pathways other than H_2_O_2_ consumption. This possibility is considered explicitly in the interpretation of the experimental results presented below. The present group previously demonstrated that BR dynamics are able to distinguish structurally similar but catalytically differentiated insoluble oxide materials [22]; here we apply this approach for the first time to a binary cobalt–molybdenum oxide. To ensure that the band-area ratios ρ_α_ are comparable, the Mo–O stretching region (≈870–1000 cm^−1^) of the ATR-FTIR spectra was deconvoluted with the same Voigt line shape for all three samples. Cyclic voltammetry (CV) and electrochemical impedance spectroscopy (EIS) provide complementary electrochemical data on the same three samples.

The central finding of this study, demonstrated for this series of three GNP-derived CoMoO_4_ samples, is that the 450 °C powder, classified as single-phase *β*-CoMoO_4_ by powder XRD, outperforms the bulk-*α* 1000 °C powder in all kinetic and electrochemical tests. This apparent paradox is partly rationalized by an empirical, semi-quantitative descriptor *Φ* = *ρ_α_* × *S_BET_*, where ρ_α_ is the relative FTIR band-area fraction of *α*-type (octahedral) Mo–O environments (a coordination-sensitive descriptor rather than a direct measurement of Lewis acidity) and *S_BET_* is the BET specific surface area. High-temperature sintering at 1000 °C completes the conversion to octahedral Mo^6+^ but strongly reduces the porosity and lowers *S_BET_*, whereas mild calcination at 450 °C yields the finest, defect-rich crystallites together with the highest α-type band-area fraction in the series, yielding the largest *Φ*. *Φ* does not, however, discriminate between the as-synthesized and 1000 °C powders, whose activities differ about fourfold; its limitations are discussed in Section 3.5. The bulk phase label alone is therefore neither necessary nor sufficient to predict peroxidase-mimetic reactivity within this three-sample series. The same powders were previously shown to differ in photocatalytic activity, the 1000 °C powder being the most efficient photocatalyst [6]; the present study addresses a different property, their reactivity towards H_2_O_2_.

## 2. Materials and Methods

### 2.1. Synthesis

The CoMoO_4_ nanopowders were prepared according to the glycine nitrate process, as described in detail in ref. [6]. Co(NO_3_)_2_·6H_2_O and (NH_4_)_6_Mo_7_O_24_·4H_2_O (Sigma-Aldrich/Merck, Darmstadt, Germany) were all dissolved together using glycine (Fischer Scientific, Waltham, MA, USA) in deionized water at the stoichiometric fuel-to-oxidizer equivalence ratio (*φ* = 1), following propellant chemistry conventions [23]. The self-propagating combustion reaction produced a highly porous as-synthesized powder. Portions were then calcined in a Nabertherm muffle furnace in static air for 10 min at 450 °C and at 1000 °C. Both temperatures lie above the *β*-CoMoO_4_ crystallization event (360–416 °C) [6], and they lie below and above, respectively, the DTA event at ≈913 °C associated with the *β* → *α* conversion of these powders [6]; the 450 °C powder therefore remains *β*-CoMoO_4_, whereas the 1000 °C powder is fully converted to *α*-CoMoO_4_.

### 2.2. Sample Characterization

**FTIR spectroscopy.** ATR-FTIR spectra were recorded on a Perkin-Elmer spectrometer equipped with a universal attenuated total reflectance (ATR) accessory over the spectral range 400–4000 cm^−1^ (20 scans, spectral resolution 4 cm^−1^); the powders were measured directly in contact with the ATR crystal, without any sample preparation. At the Mo–O stretching frequencies, the evanescent wave penetrates approximately 0.5–2 μm into the sample, so the spectra reflect predominantly the bulk and near-surface region of the particles rather than the outermost surface layer. Because this probing depth greatly exceeds the crystallite size (5–38 nm), the FTIR data describe the local coordination of whole particles rather than of their surface alone. The Mo–O stretching region (≈870–1000 cm^−1^) was deconvoluted using Voigt (Gaussian–Lorentzian convolution) profiles in area form (PeakFit, “Voigt Area” function); the same line shape was applied to all three samples. The fit itself was performed over the 500–1000 cm^−1^ window, with the same window and baseline treatment for all samples, and ρ_α_ was obtained from the components within the Mo–O stretching region. The Voigt profile was chosen because vibrational bands of solids are broadened both inhomogeneously (Gaussian contribution, from the distribution of local environments) and homogeneously (Lorentzian contribution, from finite phonon lifetimes), so that neither a pure Gaussian nor a pure Lorentzian profile describes them adequately. Within this region, the lower-wavenumber component was assigned to octahedrally coordinated Mo (*α*-type, *ν_α_*) and the higher-wavenumber component to tetrahedrally coordinated Mo (*β*-type, *ν_β_*) [14,24,25]. The relative *α*-type band-area fraction was calculated as *ρ_α_* = A(*ν_α_*)/[A(*ν_α_*) + A(*ν_β_*)], using the analytic area of each fitted band as A. For the as-synthesized sample, the *β*-type envelope was resolved into two components (948.0 and 968.2 cm^−1^), whose areas were summed. Uncertainties of ρ_α_ were propagated from the standard errors of the fitted band areas. Fits started from different initial band positions, widths and heights converged to the same band positions and areas, and the residuals showed no systematic structure. Because the *α*- and *β*-type bands lie within ~40 cm^−1^ of each other, the wavenumber dependence of the ATR penetration depth (*d_p_* ∝ 1/*ν*) changes their area ratio by less than ~5%.

Deconvolution was performed to resolve overlapping tetrahedral (*β*-type) and octahedral (*α*-type) Mo–O components that cannot be distinguished by visual inspection of the raw spectra. *ρ_α_* is a semi-quantitative relative band-area fraction. It is not an absolute phase fraction, because the IR absorption strengths of the two coordination types may differ, and it is not a direct measure of Lewis acid site density. It is used only to compare trends between samples measured under identical conditions, and it forms one factor of the comparative descriptor *Φ* = *ρ_α_* × *S_BET_* (Section 3.5). The BET surface areas and FESEM images were obtained as described in ref. [6].

**Briggs–Rauscher oscillatory reaction.** All BR experiments were conducted in a thermostated double-walled glass cell (Metrohm EA 876-20, 25 mL) at 25.0 ± 0.1 °C under continuous stirring at 900 rpm. A Pt working electrode and a Ag/AgCl (3 mol dm^−3^ KCl) reference electrode recorded the potential–time response. All reagents were analytical grade (Merck). The initial concentrations were as follows: [CH_2_(COOH)_2_]_0_ = 0.0789 mol dm^−3^, [MnSO_4_]_0_ = 0.00752 mol dm^−3^, [HClO_4_]_0_ = 0.03 mol dm^−3^, [KIO_3_]_0_ = 0.0752 mol dm^−3^, [H_2_O_2_]_0_ = 1.176 mol dm^−3^. The reagents were added in the following fixed order: (1) CH_2_(COOH)_2_, (2) MnSO_4_, (3) HClO_4_, and (4) KIO_3_. All CoMoO_4_ samples were introduced as a suspension 3 min prior to H_2_O_2_ addition, which defined t_0_ = 0. The oscillatory time *τ_osc_* was taken as the interval from H_2_O_2_ addition to the last observable oscillation. The mass ranges tested were as follows: 0.0103–0.0400 g (as-synthesized), 0.0011–0.0074 g (450 °C), 0.0515–0.1500 g (1000 °C). All BR experiments were performed in triplicate (n = 3) for each catalyst mass. tThe vertical error bars are 95% confidence intervals of *τ_osc_* calculated from Student’s t-distribution (±5 s), and the horizontal error bars correspond to the uncertainty of the catalyst mass (±0.0001 g). The regression parameters of the calibration lines are given with their standard errors. Because oscillation suppression is a yes/no outcome, the suppression threshold is reported as the mass interval within which suppression occurred in all replicates.

The mechanistic basis for using *τ_osc_* as a kinetic probe of H_2_O_2_-related reactivity lies in the central role of H_2_O_2_ in the BR mechanism: H_2_O_2_ reduces iodate and hypoiodous acid to iodide and regenerates Mn^2+^ from Mn^3+^ in the radical, autocatalytic pathway, so that any solid that heterogeneously decomposes H_2_O_2_ withdraws a key reactant from the oscillator and shortens *τ_osc_* [18,19,20]. The sensitivity of *τ_osc_* to H_2_O_2_ perturbation arises from the non-linearity of the autocatalytic feedback, as established by independent mechanistic and kinetic studies of the BR system [18,20,21]. The application of this framework to discriminate the H_2_O_2_ reactivity of insoluble solid oxide materials was previously demonstrated by the present group [22]. It should be noted that dissolved transition-metal species released from the solid surface—including Co^2+^ ions and Mo-oxo complexes—may also perturb the BR oscillatory dynamics through pathways other than H_2_O_2_ consumption. This possibility is considered explicitly in the interpretation of the results in Section 3.3.

**Cyclic voltammetry and EIS.** All electrochemical measurements used a three-electrode cell containing a Pt disc working electrode (geometric area 0.07 cm^2^), a Pt rod counter electrode, and a Ag/AgCl (3 mol dm^−3^ KCl) reference electrode (+0.210 V vs. NHE), driven by an Autolab PGSTAT302N potentiostat (Metrohm, The Netherlands) under NOVA 2.1. CV was recorded at 50 mV s^−1^ in four electrolytes (25 mL each; background 0.03 mol dm^−3^ HClO_4_): malonic acid (0.0789 mol dm^−3^), MnSO_4_ (0.00752 mol dm^−3^), KIO_3_ (0.0752 mol dm^−3^) and H_2_O_2_ (1.176 mol dm^−3^), both with and without 0.02 g of each CoMoO_4_ sample. The scan-rate-dependent measurements (20–300 mV s^−1^) were carried out in the H_2_O_2_ + HClO_4_ electrolyte; CV in this electrolyte is the electrochemical measurement performed under reaction-relevant conditions. The EIS was recorded in 0.2 mol dm^−3^ KCl at an open-circuit potential (100 kHz–10 mHz; 10 mV rms perturbation). In order to characterize the interfacial properties of the electrode–powder suspension in the absence of faradaic reactions from the oscillatory reagents, the EIS measurements were performed in KCl rather than under H_2_O_2_ reaction conditions; therefore, the relationship between the derived impedance parameters and peroxidase-mimetic activity is interpreted as correlative rather than mechanistic (see Section 3.4). The Nyquist plots were fitted to the Randles equivalent circuit *R_s_*(*Q*(*R_ct_*)) using the NOVA 2.1 software (Metrohm Autolab, Utrecht, The Netherlands). The effective double-layer capacitance was extracted via the Brug model [26].

## 3. Results and Discussion

### 3.1. Bulk Structure, Morphology, and Textural Properties

Powder XRD confirms single-phase *β*-CoMoO4 (*C2*/*m*, ICSD #78328; tetrahedral Mo^6+^) for the as-synthesized and 450 °C samples and single-phase *α*-CoMoO_4_ (*C2*/*m*, ICSD #23808; octahedral Mo^6+^) for the 1000 °C sample (Table 1). The *β* → *α* conversion contracts the unit cell from ≈703 to ≈601 Å^3^ at the DTA endotherm near 913 °C [6], and the lattice parameters agree well with published ICSD data for both phases [6,16].

**Figure 1:**
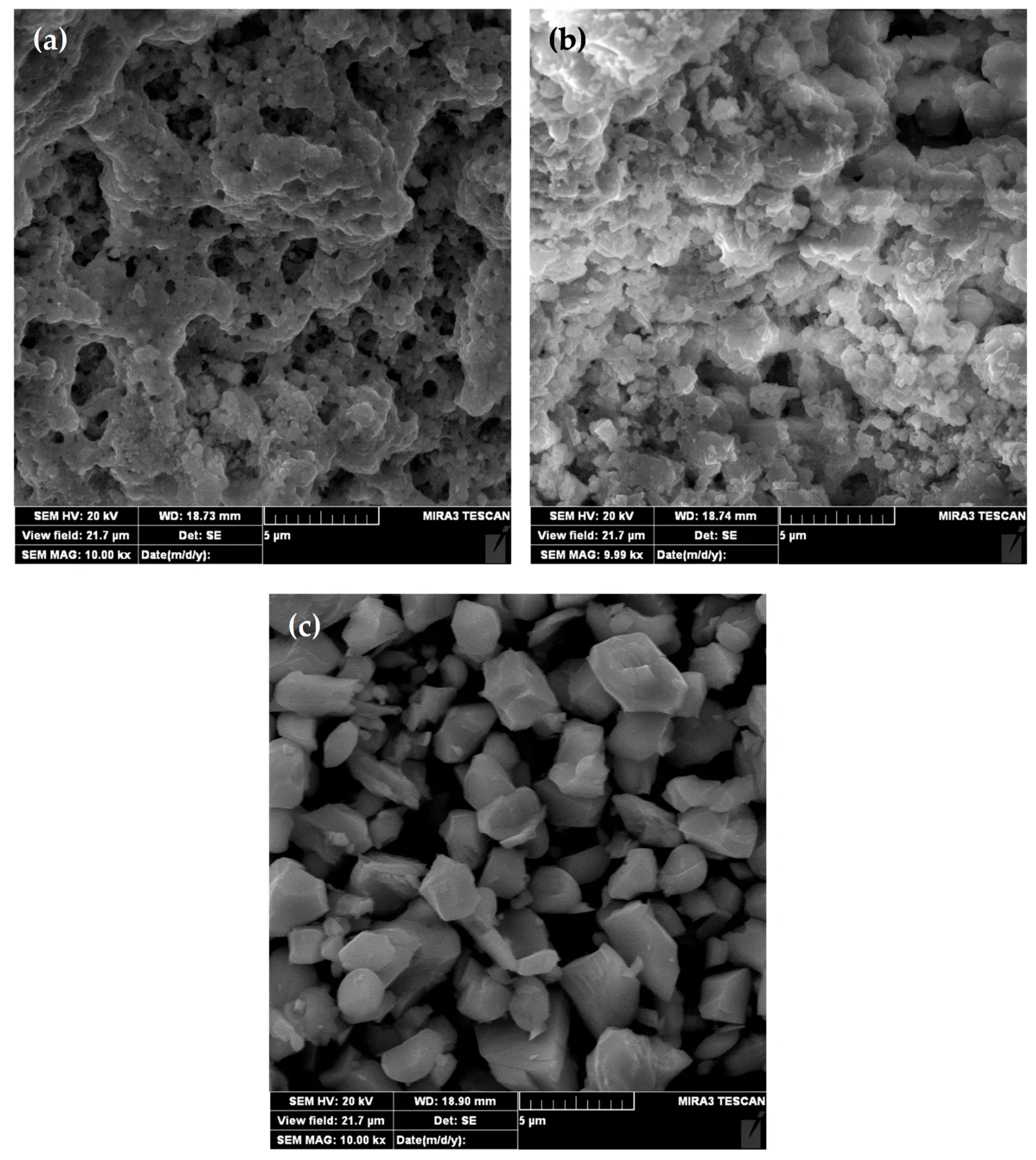
FESEM images of (**a**) CoMoO_4_ as-synthesized, (**b**) CoMoO_4_ at 450 °C, and (**c**) CoMoO_4_ at 1000 °C; scale bar 5 μm.

Calcination changes the texture systematically. The BET surface area goes from 22 m^2^ g^−1^ (as-synthesized) up to 24 m^2^ g^−1^ at 450 °C and then drops to 15 m^2^ g^−1^ at 1000 °C, while the crystallite size grows from 5.2 to 38.4 nm [6]. *S_BET_* is used here as a measure of the textural surface area; its accessibility to H_2_O_2_ under reaction conditions is an assumption. FESEM (Figure 1) shows the corresponding morphological progression: a highly porous sponge-type network for the as-synthesized powder, formed by the large gas volume released during combustion, which also retains residual amorphous and hydroxylated surface phases from the incompletely crystallized combustion product; grape-like agglomerates of fine crystallites with interstitial voids for the 450 °C powder; and well-faceted polyhedral grains for the 1000 °C powder, with no open porosity resolved at FESEM magnification.

### 3.2. FTIR Spectroscopy: Local Coordination Heterogeneity

**Figure 2:**
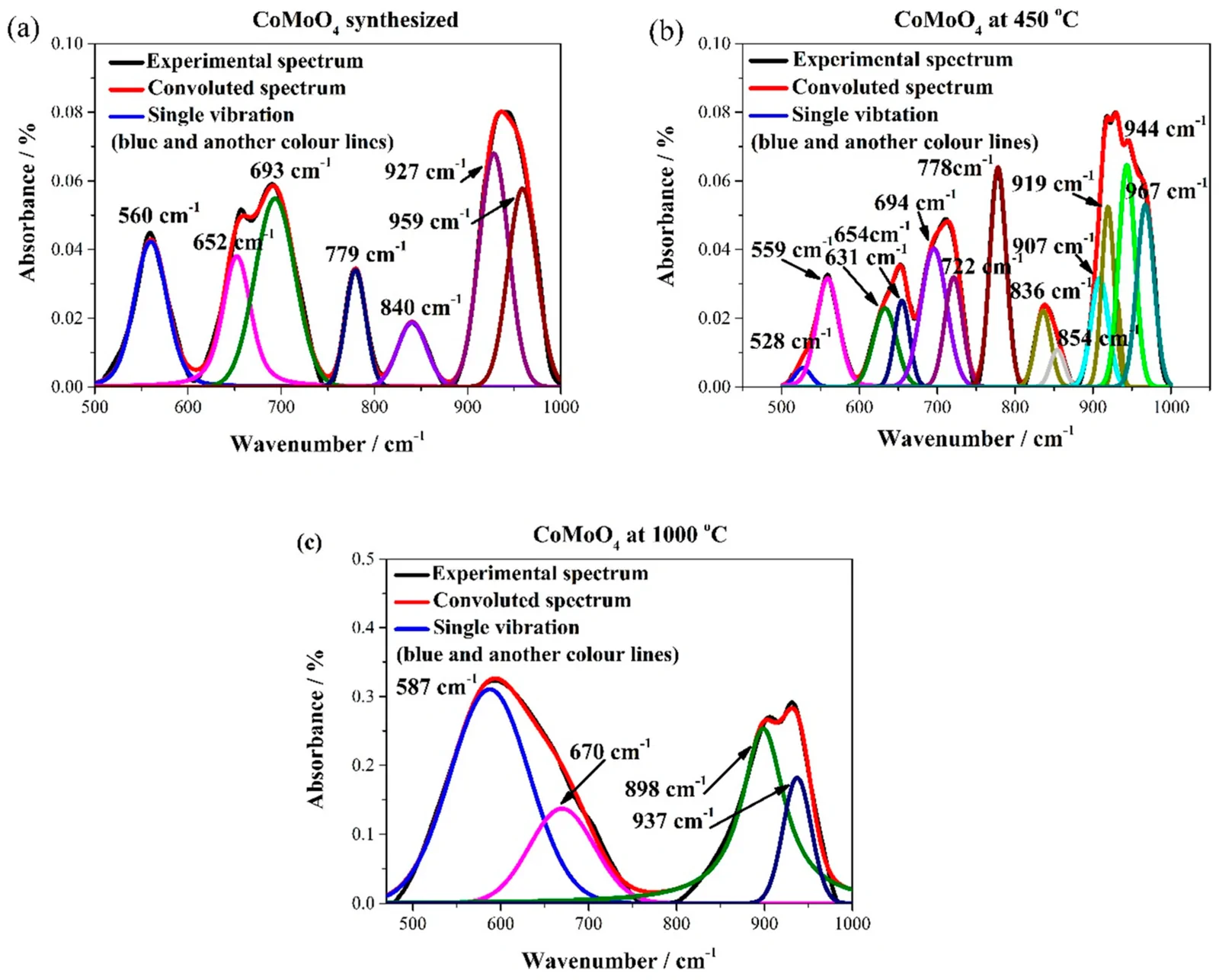
Deconvolution of the 500–1000 cm^−1^ region of the FTIR spectra of (**a**) CoMoO_4_ as-synthesized, (**b**) CoMoO_4_ at 450 °C, and (**c**) CoMoO_4_ at 1000 °C. Coloured sub-bands are the fitted Voigt components; red envelope, cumulative fit. The same line shape, fitting window and baseline treatment were used for all three spectra.

Infrared spectroscopy is sensitive to the local coordination environment of each Mo^6+^ centre rather than to long-range crystallographic order, making it the method of choice for detecting local coordination states that bulk XRD does not register. The two CoMoO_4_ polymorphs differ precisely in their Mo^6+^ local environment: *β*-CoMoO_4_ is built from isolated MoO_4_ tetrahedra, while *α*-CoMoO_4_ contains edge-sharing MoO_6_ octahedra assembled into Mo_4_O_16_ tetramers [11,24]. The two envelopes in the Mo–O stretching region are assigned on the basis of the literature on CoMoO_4_ [14,24,25] and of our own α-CoMoO_4_ reference: in the 1000 °C powder, which is single-phase α-CoMoO_4_ by XRD, 76% of the fitted Mo–O stretching area lies in the lower-wavenumber component (898.6 cm^−1^). The Hardcastle–Wachs correlation [27] relates the stretching frequency to individual Mo–O bond lengths; because the distorted MoO_6_ octahedra contain both short terminal (≈1.73 Å) and long (up to ≈2.25 Å) Mo–O bonds, this correlation alone does not order the two envelopes unambiguously and is not used here as the main argument. On this basis, within the Mo–O stretching region, the lower-wavenumber component (≈926–899 cm^−1^) is assigned to *α*-type and the higher-wavenumber component (≈968–937 cm^−1^) to *β*-type Mo^6+^ environments (Figure 2, Table 2).

Both components appear in the as-synthesized powder, which XRD classifies as single-phase *β*-CoMoO_4_ [6]: the *β*-type envelope (948.0 and 968.2 cm^−1^) arises from terminal Mo=O stretching of isolated MoO_4_ tetrahedra, and the α-type band at 925.4 cm^−1^ is attributed to distorted, octahedral-like Mo–O environments, which are not expected in a purely tetrahedral framework [11,24]. The band positions are stable with respect to the starting parameters of the fit. The 450 °C powder shows the same two contributions (925.9 and 964.4 cm^−1^), but with a much larger relative contribution of the *α*-type band. The 1000 °C powder, fully converted to *α*-CoMoO_4_ according to XRD, is dominated by a broadened *α*-type band shifted to 898.6 cm^−1^, accompanied by a weaker higher-wavenumber component at 937.3 cm^−1^.

These observations are consistent with what the CoMoO_4_ energy landscape calculation predicts [19]: multiple structural modifications exist within a narrow energy range, and the spatially irregular GNP combustion front can nucleate different local environments in adjacent microdomains. What FTIR detects is the result—a material whose local Mo–O coordination is consistent with the presence of *α*-like octahedral Mo^6+^ environments even where the bulk diffraction pattern reads as pure *β*-CoMoO_4_. The band-area ratio ρ_α_ is used here as a comparative structural descriptor of the as-synthesized and annealed (450 °C and 1000 °C) samples. Changes in ρ_α_ may be qualitatively related to changes in the coordination environment of Mo and Co cations; however, *ρ_α_* is not a direct measure of Lewis acid site density, which would require independent characterization of surface acidity.

All fits reproduce the experimental spectra well (R^2^ ≥ 0.9925; Table 3). In the as-synthesized powder, the *α*- and *β*-type contributions are comparable (*ρ_α_* = 0.47 ± 0.25); the large uncertainty arises because three strongly overlapping components (925.4, 948.0 and 968.2 cm^−1^) must be resolved within a narrow region. After annealing at 450 °C, the *α*-type band dominates the Mo–O stretching region (*ρ_α_* = 0.90 ± 0.07), with the *β*-type band accounting for ≈10% of the Mo–O stretching area. After annealing at 1000 °C, the *α*-type contribution remains dominant (ρ_α_ = 0.76 ± 0.02); the *α*-type band is shifted to 898.6 cm^−1^ and broadened, with a pronounced Lorentzian contribution, indicating a change in the local Mo–O environment and/or crystallinity. That *ρ_α_* does not reach unity even for this sample, which is single-phase *α*-CoMoO_4_ by XRD, further shows that *ρ_α_* is a relative band-area descriptor rather than a phase fraction. Within uncertainty, the *ρ_α_* values of the as-synthesized and 1000 °C powders cannot be distinguished (they differ by only about 1.2 standard uncertainties); only the 450 °C powder stands out.

The high *ρ_α_* obtained for the 450 °C powder, which is single-phase *β*-CoMoO_4_ by XRD, must be interpreted with caution. Band-area fractions are not phase fractions, and the ≈926 cm^−1^ band may also contain contributions from distorted or defective Mo–O environments and from poorly crystalline molybdate domains below the XRD detection limit. *ρ_α_* therefore indicates only the relative contribution of octahedral-type Mo–O environments within this series. It should also be noted that the *α*-type component of the two *β*-classified powders (925–926 cm^−1^) lies about 27 cm^−1^ above that of *α*-CoMoO_4_ (898.6 cm^−1^); it therefore cannot be identified with crystalline *α*-CoMoO_4_, and a contribution from modes intrinsic to the *β*-CoMoO_4_ lattice cannot be excluded. Combined with the BET surface area, it gives *Φ* = 21.5 ± 1.7 m^2^ g^−1^ for the 450 °C powder, which has both the highest *ρ_α_* and the largest *S_BET_* (24 m^2^ g^−1^). At 1000 °C, the lower *ρ_α_* and the reduced surface area (15 m^2^ g^−1^) give *Φ* = 11.4 ± 0.3 m^2^ g^−1^, which is indistinguishable within uncertainty from that of the as-synthesized powder (10.3 ± 5.5 m^2^ g^−1^).

### 3.3. Briggs–Rauscher Oscillatory Reaction: A Quantitative Kinetic Probe

An autocatalytic iodine feedback loop sustains the BR oscillatory reaction [16,17]: H_2_O_2_ acts mainly as the reductant that converts iodate and hypoiodous acid to iodide and regenerates Mn^2+^ from Mn^3+^ in the radical, autocatalytic pathway [18,20]. The Pt electrode monitors the overall oscillatory redox potential, cycling between ∼790 mV (I_2_-dominated oxidized state) and ∼690 mV (I^−^-dominated reduced state) [22]. Any solid surface that decomposes H_2_O_2_ withdraws this reactant from the oscillator and shortens *τ_osc_*. CoMoO_4_ has been proposed to act as a peroxidase mimic via Co^2+^/Co^3+^ redox cycling facilitated by surface Mo^6+^ sites that, in octahedral-like coordination, are expected to exhibit Lewis-acidic character [8]. The non-linearity of the BR feedback amplifies differences in the H_2_O_2_ consumption rate into qualitatively distinct macroscopic outcomes, making it an unusually sensitive kinetic probe of reactivity towards H_2_O_2_. A contribution of dissolved species to the BR response cannot be completely excluded (Section 2.2).

#### 3.3.1. CoMoO_4_ As-Synthesized: Linear Mass Dependence and Gravimetric Calibration

**Figure 3:**
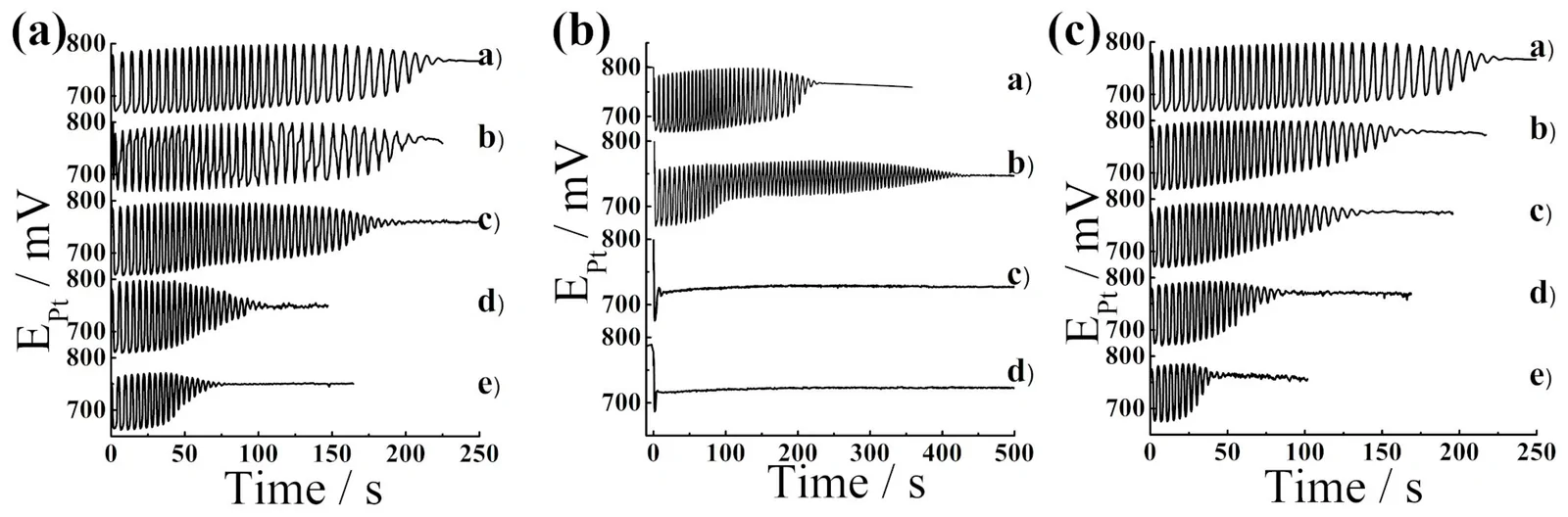
(**a**) Potential–time oscillograms (E_Pt_ vs. Ag/AgCl) for the BR reaction without CoMoO_4_ as-synthesized (a) and with 0.0103 g (b), 0.0175 g (c), 0.0300 g (d), and 0.0400 g (e). T = 25.0 °C, 900 rpm. The preserved oscillogram morphology is consistent with a perturbation that mainly shortens the oscillatory regime; (**b**) BR oscillograms without CoMoO_4_ at 450 °C (a) and with 0.0011 g (b), 0.0020 g (c), and 0.0074 g (d). Trace (b): extension of *τ_osc_* with compressed amplitude, attributed to the non-linear response of the oscillator to the added solid (see text). Traces (c) and (d): complete oscillation suppression. The suppression threshold (0.0011–0.0020 g, n = 3) is at least 20-fold lower than for CoMoO_4_ as-synthesized; (**c**) BR oscillograms without CoMoO_4_ at 1000 °C (a) and with 0.0515 g (b), 0.0753 g (c), 0.1000 g (d), and 0.1500 g (e). T = 25.0 °C. Qualitatively analogous to CoMoO_4_ as-synthesized but requiring ≈4× more mass, consistent with its smaller textural surface area and strongly reduced porosity.

**Figure 4:**
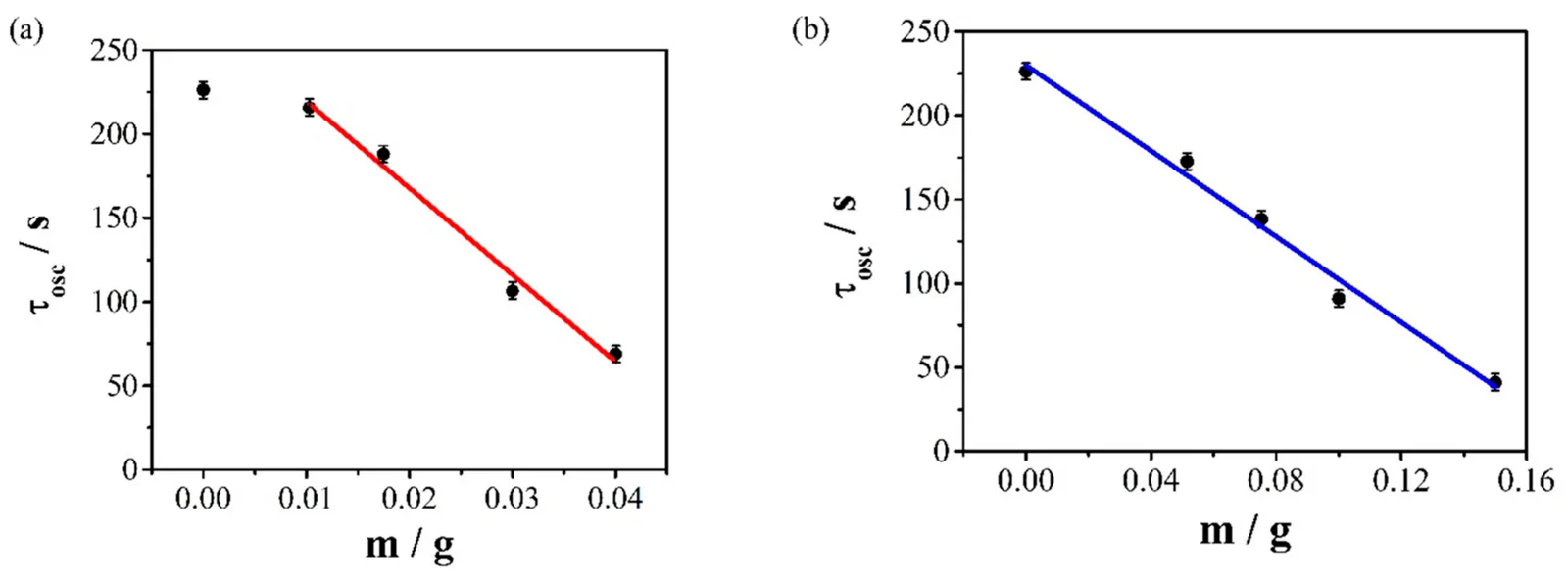
(**a**) Oscillatory time *τ_osc_* vs. mass of CoMoO_4_ as-synthesized; solid line: linear fit, Equation (1), adj. R^2^ = 0.982. (**b**) *τ_osc_* vs. mass of CoMoO_4_ at 1000 °C; solid line: linear fit, Equation (2), adj. R^2^ > 0.98. Points are means of three measurements (n = 3); vertical error bars are 95% confidence intervals (Student’s t-distribution) and horizontal error bars the uncertainty of the catalyst mass (±0.0001 g). The slope for the as-synthesized powder is 4.05 ± 0.41 times that for the 1000 °C powder.

BR oscillograms for the blank (trace a) and CoMoO_4_ as-synthesized at 0.0103 g (b), 0.0175 g (c), 0.0300 g (d), and 0.0400 g (e) are shown in Figure 3a. The blank shows regular oscillations (∼100 mV amplitude; *τ_osc_* ≈ 250 s). Addition of CoMoO_4_ as-synthesized reduces *τ_osc_* monotonically while fully preserving oscillogram morphology: amplitude and waveform shape remain unchanged at all masses, which is consistent with a perturbation that mainly shortens the oscillatory regime without a marked change in iodine speciation. For each experimental point, three replicate measurements of *τ_osc_* were performed, and the uncertainty of *τ_osc_* was determined using Student’s t-distribution at a 95% confidence level. Figure 4a presents the dependence of *τ_osc_* on mass, together with the corresponding error bars. The mass dependence is linear (Figure 4a):

(1) *τ_osc_* = (271.3 ± 10.9) − (5172 ± 406) × *m*       [adj. R^2^ = 0.982]

where *τ_osc_* is in seconds and *m* is mass in grams; the uncertainties are standard errors of the regression parameters. Linearity over 0.01–0.04 g is consistent with a constant mass-specific effect across this mass range. Equation (1) simultaneously provides a gravimetric analytical calibration function with a sensitivity of 5172 ± 406 s g^−1^.

#### 3.3.2. CoMoO_4_ at 450 °C: Complete Oscillation Suppression at Milligram Loading

CoMoO_4_ at 450 °C produces qualitatively and quantitatively distinct BR behaviour (Figure 3b), which constitutes the most striking kinetic result in this study. At 0.0011 g (trace b), *τ_osc_* is extended to ∼350 s instead of being shortened, oscillatory amplitude is compressed to ∼30 mV centred near 745 mV, and the waveform becomes irregular and asymmetric. This counterintuitive behaviour is a consequence of the non-linear nature of the BR oscillatory system combined with specific properties of the material itself. A synergistic effect may also play a role: in non-linear systems, even small perturbations can shift the system in phase space, and the response to an added heterogeneous (insoluble) material is therefore not a simple sum of the individual contributions. We have previously observed comparable behaviour with clay minerals [28], and in that case, the effect likewise could not be attributed solely to the leaching of ions into the reaction mixture. A possible contribution from Co and Mo ions leached from the powder cannot be completely excluded; however, because the three samples have the same chemical composition but affect the oscillator very differently, their structural and surface characteristics are supposed to be the dominant factor. It should be emphasized that this interpretation is proposed as a working hypothesis pending direct characterization of solution speciation. The amplitude compression and waveform distortion in this work confirm a substantial mechanistic perturbation even at this low mass (1.1 mg) of CoMoO_4_ at 450 °C.

At 0.0020 g (trace c) and 0.0074 g (trace d), oscillations are completely suppressed: E_Pt_ drops to ≈720 mV within seconds of H_2_O_2_ addition, indicating that the H_2_O_2_ concentration has decreased substantially, most likely below the level required to sustain the oscillatory regime. However, the potential alone does not allow for a definitive conclusion regarding the complete consumption of H_2_O_2_. The suppression threshold of CoMoO_4_ at 450 °C lies between 0.0011 and 0.0020 g in all three replicates, at least 20-fold lower than the mass at which CoMoO_4_ as-synthesized remains oscillatory (0.040 g). Since both samples share identical elemental composition, this gap most likely originates in structural, morphological and surface differences. We attribute it to three factors acting together in CoMoO_4_ at 450 °C: (i) the largest textural surface area per gram in the series (*S_BET_* = 24 vs. 22 m^2^ g^−1^ for CoMoO_4_ as-synthesized, a modest 9% difference); (ii) the smallest crystallite size in the series (D = 5.2 nm, Table 1), with the crystallites assembled into grape-like agglomerates with interstitial voids (Figure 1b), which exposes a high density of crystallite edges and grain boundaries to the reaction medium;; and (iii) the highest relative contribution of octahedral-type Mo–O environments in the series (*ρ_α_* = 0.90 ± 0.07; Section 3.2), which, on structural grounds, may provide more reactive Mo^6+^ sites than the tetrahedral Mo^6+^ of the *β*-CoMoO_4_ bulk. Factor (i) alone cannot explain the >20-fold reactivity difference, because the surface area gap is far too small. The combination of fine crystallite morphology and local Mo coordination is the most plausible explanation within this series, although contributions from factors not measured here (Co^2+^/Co^3+^ redox chemistry, oxygen vacancies, surface hydroxylation) and from leached ions cannot be completely excluded.

#### 3.3.3. CoMoO_4_ at 1000 °C: Moderate Reactivity Despite Bulk α-CoMoO_4_ Phase

CoMoO_4_ at 1000 °C shows progressive, morphologically smooth *τ_osc_* reduction without oscillation suppression over 0.0515–0.1500 g (Figure 3c and Figure 4b):

(2) *τ_osc_* = (230.1 ± 7.3) − (1278 ± 80) × *m*       [adj. R^2^ > 0.98]

The slope ratio (CoMoO_4_ as-synthesized)/(CoMoO_4_ at 1000 °C) = 5172/1278 = 4.05 ± 0.41: CoMoO_4_ as-synthesized is approximately four times more active per gram than CoMoO_4_ at 1000 °C; the difference between the two slopes is about nine times its combined standard error. This ordering is initially surprising, because CoMoO_4_ at 1000 °C is bulk *α*-CoMoO_4_ with octahedral Mo^6+^ centres expected, on structural grounds, to be more Lewis-acidic, while CoMoO_4_ as-synthesized is bulk *β*-CoMoO_4_ with tetrahedral Mo^6+^. Two factors are likely to contribute. First, CoMoO_4_ at 1000 °C has a 32% lower BET surface area (15 vs. 22 m^2^ g^−1^) and hence a smaller textural surface area per gram. Second, its smooth morphology with strongly reduced porosity (Figure 1c) is likely to restrict substrate access to whatever surface *α*-CoMoO_4_ sites are present. The macroscopic rate per gram is therefore determined by the product of intrinsic per-site activity and geometric accessibility, and for CoMoO_4_ at 1000 °C, the latter appears to be restricted by calcination to such an extent that any advantage in per-site activity expected for bulk *α*-CoMoO_4_ is more than offset. This motivates the *Φ* = *ρ_α_ × S_BET_* descriptor discussed in Section 3.5.

### 3.4. Cyclic Voltammetry and Electrochemical Impedance Spectroscopy

**Figure 5:**
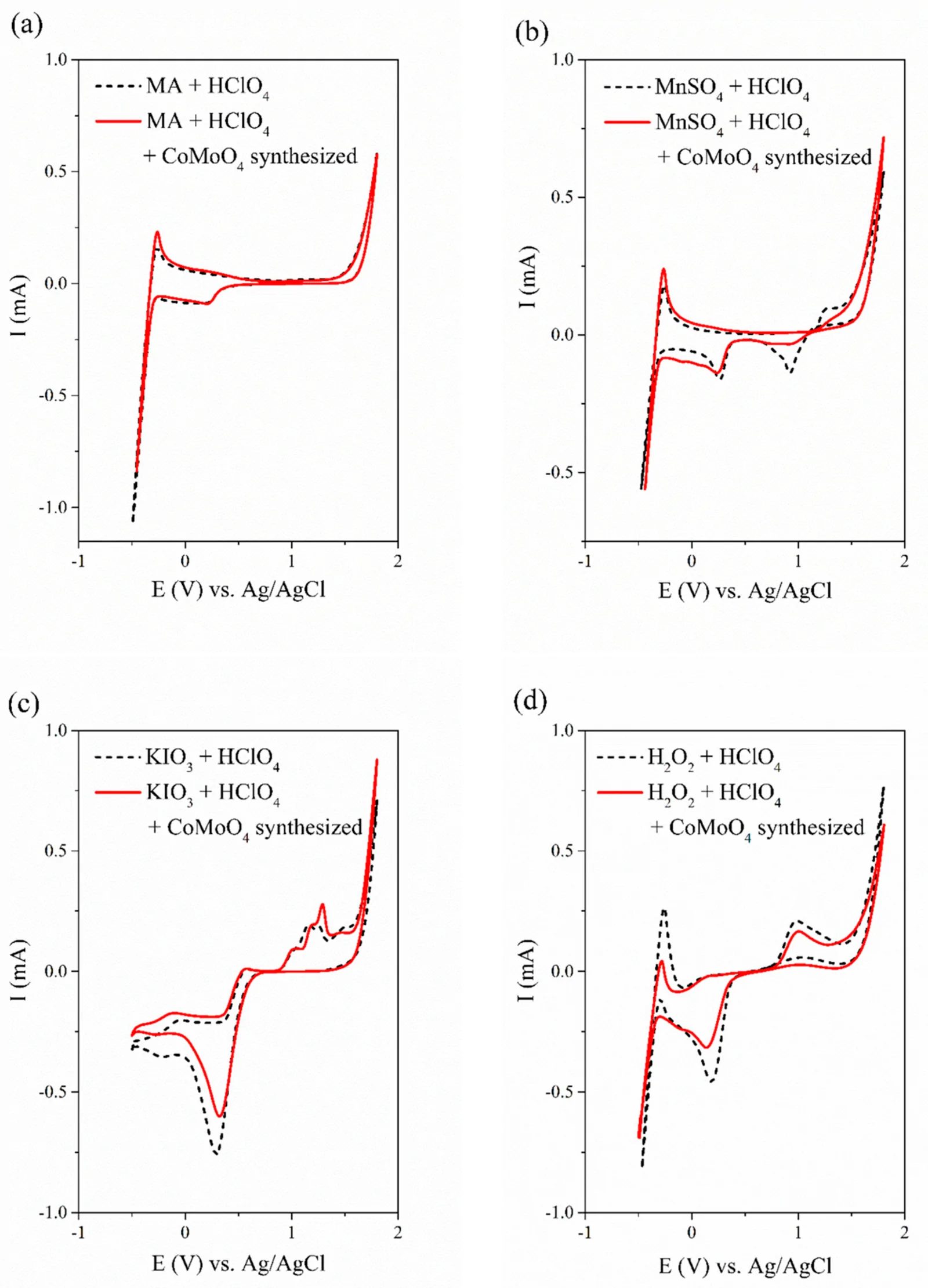
Cyclic voltammograms recorded in the (**a**) MA–HClO_4_; (**b**) MnSO_4_–HClO_4_; (**c**) KIO_3_–HClO_4_, and (**d**) H_2_O_2_–HClO_4_ electrolytes without (dashed) and with (solid) the added synthesized CoMoO_4_ powders.

The cyclic voltammograms recorded for the investigated set of electrolytes, with and without added samples of synthesized CoMoO_4_, are presented in Figure 5a–d. A clear difference in the voltammetric response was observed in the MnSO_4_- and H_2_O_2_-containing electrolytes for the as-synthesized CoMoO_4_ and the CoMoO_4_ at 450 °C samples (Figure 5; for CoMoO_4_ at 450 °C, see also Figure S1a–d), whereas the CoMoO_4_ at 1000 °C sample (Figure S2a–d) produced only a minor perturbation, confined to the H_2_O_2_-containing system.

The close similarity of the response between CoMoO_4_ as-synthesized and CoMoO_4_ at 450 °C is consistent with their shared bulk β-CoMoO_4_ structure and comparable surface areas, although the two powders differ markedly in the BR test (Section 3.3). Phase-dependent differences in the interaction of CoMoO_4_ polymorphs with oxygen-containing molecules such as water and H_2_O_2_ have been reported previously [29]: preferential interaction of water with α-CoMoO_4_ has been ascribed to electronically less dense surface regions that favour binding of oxygen, while the deficiency in oxygen atoms on specific crystallographic planes explains the preferential interaction of certain CoMoO_4_ surfaces with H_2_O_2_ [29].

**Figure 6:**
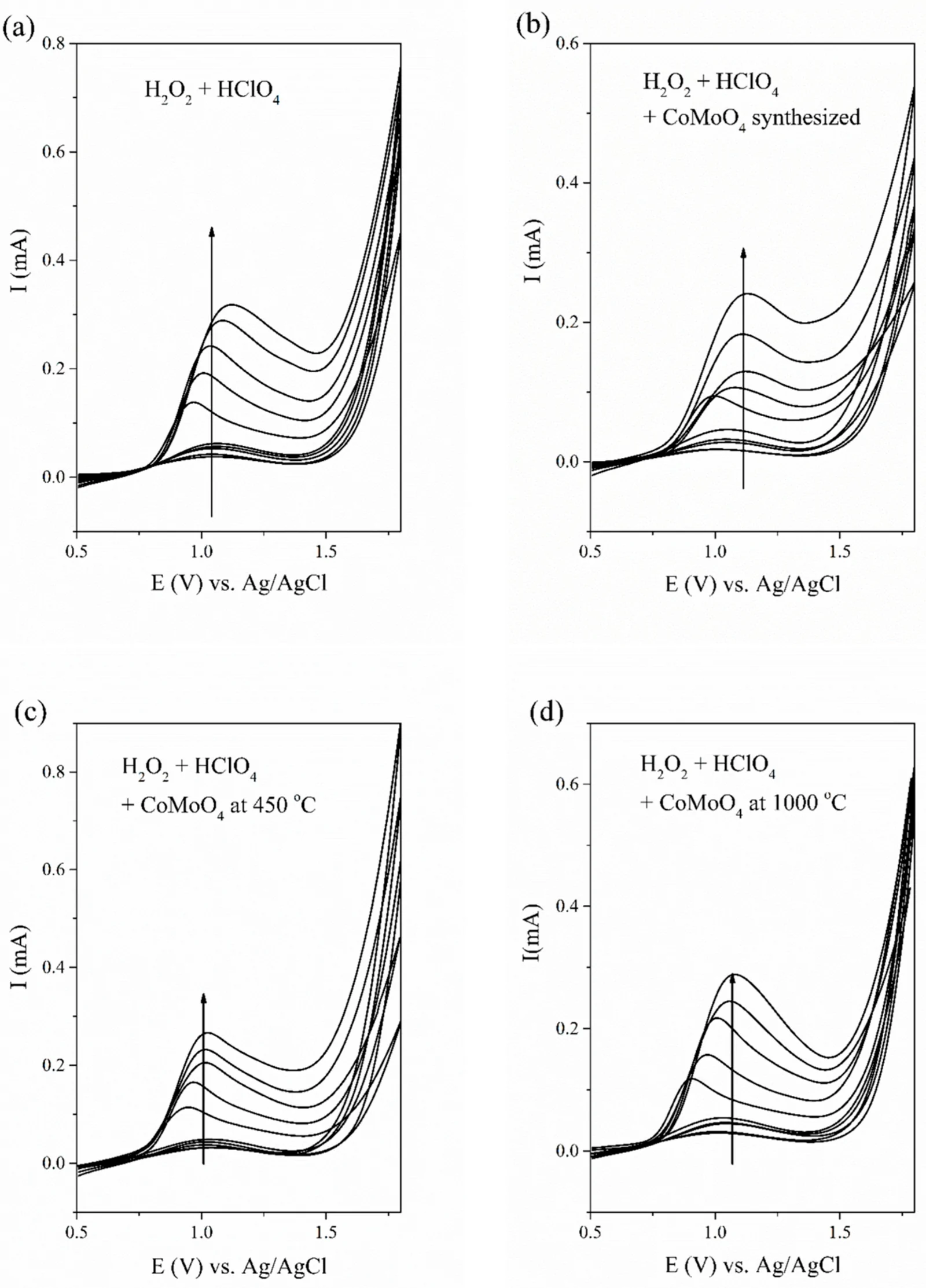
Cyclic voltammograms recorded at different scan rates in the H_2_O_2_–HClO_4_ electrolyte: (**a**) blank, and with the added CoMoO_4_ sample: (**b**) CoMoO_4_ as-synthesized, (**c**) CoMoO_4_ at 450 °C and (**d**) CoMoO_4_ at 1000 °C.

Since all three samples influenced the H_2_O_2_ oxidation process, the voltammetric behaviour was examined further as a function of scan rate (Figure 6a–d).

**Figure 7:**
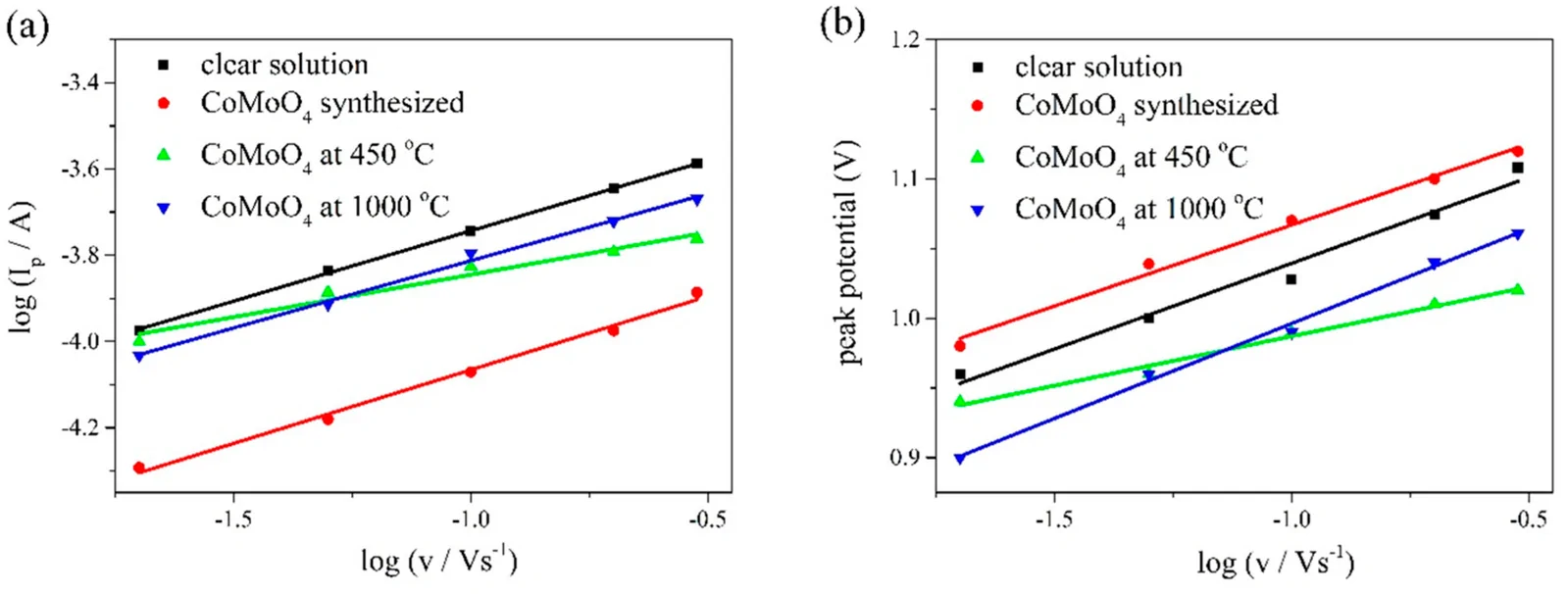
(**a**) Dependence of log(*I*_p_) on log(*v*) and (**b**) dependence of the peak potential *E*_p_ on log(*v*) for the blank solution and for the electrolytes containing the CoMoO_4_ samples.

The slopes of the log(*I*_p_) vs. log(ν) plots (Figure 7a) amounted to 0.33, 0.34, 0.20, and 0.31 for the blank solution and for the electrolytes containing CoMoO_4_ as-synthesized, CoMoO_4_ at 450 °C, and CoMoO_4_ at 1000 °C, respectively. Theoretically, a slope of 0.5 is expected for a purely diffusion-controlled process and 1 for a purely adsorption-controlled process, with intermediate values indicating mixed control; values below 0.5 signal an additional kinetic (chemical) contribution to the overall rate [30]. The comparatively low slope of the blank (0.33) is consistent with the complex autocatalytic decomposition of H_2_O_2_ at platinum in acidic media and the concomitant formation of adsorbed oxygen species at the Pt surface, and it is taken as the effective baseline for the system. The pronounced decrease to 0.20 for CoMoO_4_ at 450 °C indicates that this material further modifies the H_2_O_2_ reaction mechanism.

A plausible interpretation of this behaviour is a chemical–electrochemical (CE) mechanism, in which a heterogeneous catalytic step precedes electron transfer [30,31]. The highly active surface of CoMoO_4_ at 450 °C drives the heterogeneous catalytic decomposition of H_2_O_2_ within the bulk solution and the diffusion layer, depleting the electroactive species before it reaches the Pt surface. At high scan rates, the short experimental timescale limits this prior depletion, and the diffusion layer remains relatively undisturbed; at low scan rates, the longer timescale allows the catalyst to substantially deplete the local H_2_O_2_ concentration near the electrode. The resulting selective suppression of the peak current at low scan rates compresses the overall current range across the scan-rate window, directly lowering the apparent slope to 0.20.

The slopes of the *E_p_* vs. log(*ν*) plots (Figure 7b) followed the same trend. The corresponding slopes were 123, 116, 71, and 136 mV dec^−1^ for the blank and for CoMoO_4_ as-synthesized, CoMoO_4_ at 450 °C, and CoMoO_4_ at 1000 °C, respectively. For a totally irreversible, diffusion-controlled process, the peak potential varies with scan rate according to [25,26]:

(3) *E_p_* = (*b*/2) log *ν* + *const.*

where *b* = 2.303*RT*/(*αnF*) = 0.059/(*αn*) V at 25 °C is the Tafel slope, with *α* being the transfer coefficient and *n* the number of electrons transferred. The experimentally measured *∂E_p_*/*∂*log *ν* therefore corresponds to *b*/2, so the apparent Tafel slope for CoMoO_4_ at 450 °C is *b* ≈ 142 mV dec^−1^ (from *∂E*_p_/*∂*log ν = 71 mV dec^−1^). Because the system operates under mixed kinetic control, however, these apparent slopes cannot be used for reliable extraction of α and *n*; they are reported here only as a qualitative, comparative diagnostic.

**Figure 8:**
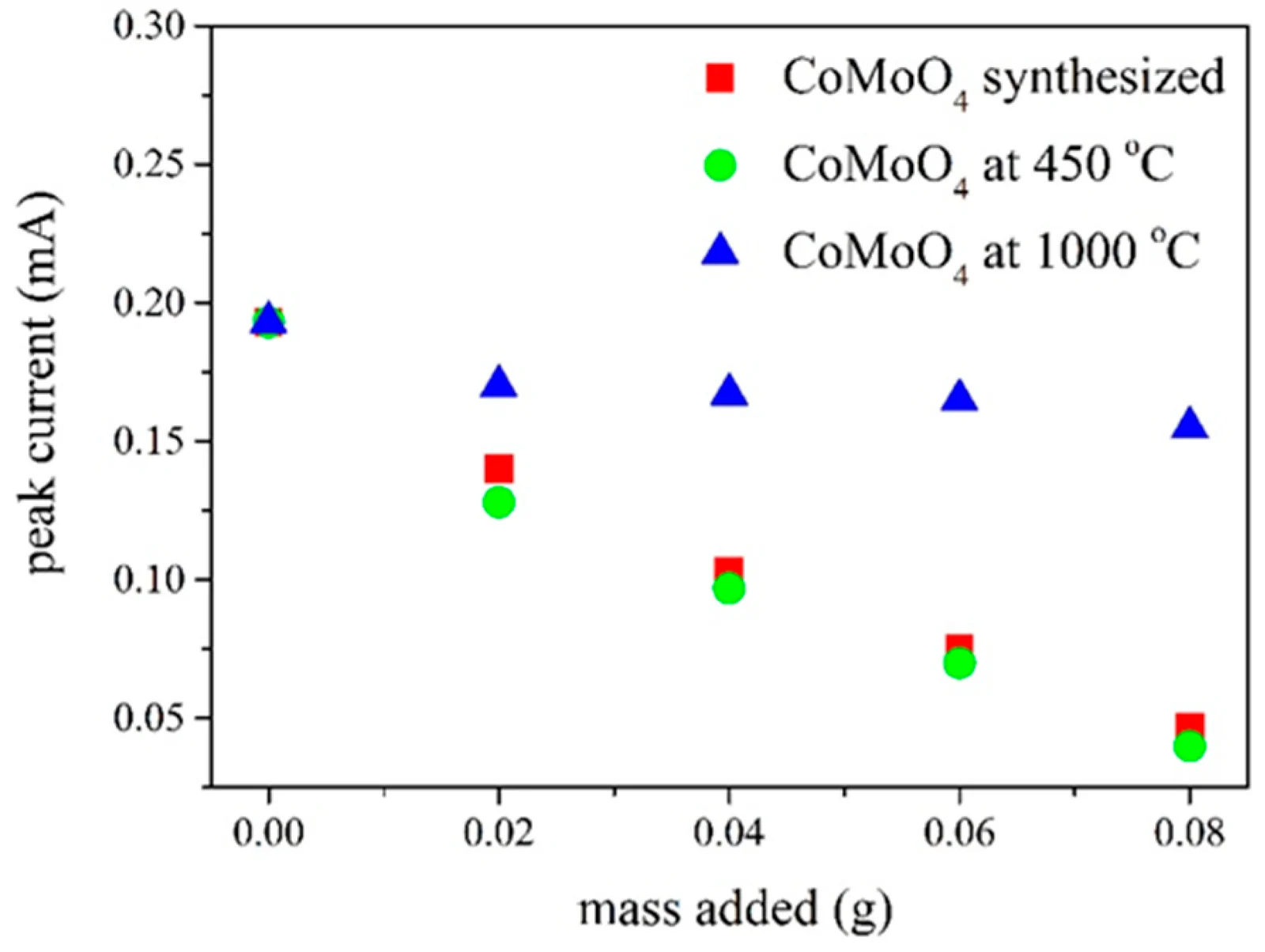
Dependence of the peak current on the mass of CoMoO_4_ added to the H_2_O_2_–HClO_4_ electrolyte.

The influence of catalyst loading was assessed by recording a voltammogram after each successive addition of a sample aliquot (Figure 8). CoMoO_4_ as-synthesized and CoMoO_4_ at 450 °C produced a comparable, marked decrease in peak current, whereas CoMoO_4_ at 1000 °C had a much more moderate effect. This trend follows the activity ranking obtained in the BR test and is attributed to competitive heterogeneous H_2_O_2_ disproportionation at the powder surface:

(4) 2 H_2_O_2_    →    2 H_2_O + O_2_

The 1000 °C sample, despite being bulk *α*-CoMoO_4_, is far less active in this respect, most likely because calcination has reduced both the accessible surface area and the porosity available to the substrate. Although CoMoO_4_ as-synthesized also lowers the absolute peak current—indicating that it also decomposes H_2_O_2_—it retains a log(*I*_p_)/log(*ν*) slope of 0.34, essentially identical to that of the blank (0.33). Within the CE framework, this divergence reflects the magnitude of the heterogeneous rate constant: CoMoO_4_ at 450 °C acts as a fast catalyst that dynamically perturbs the transient diffusion layer during the voltammetric sweep, while the slower CoMoO_4_ as-synthesized depletes the bulk concentration without appreciably altering the diffusion layer on the experimental timescale, keeping the slope at 0.34.

**Figure 9:**
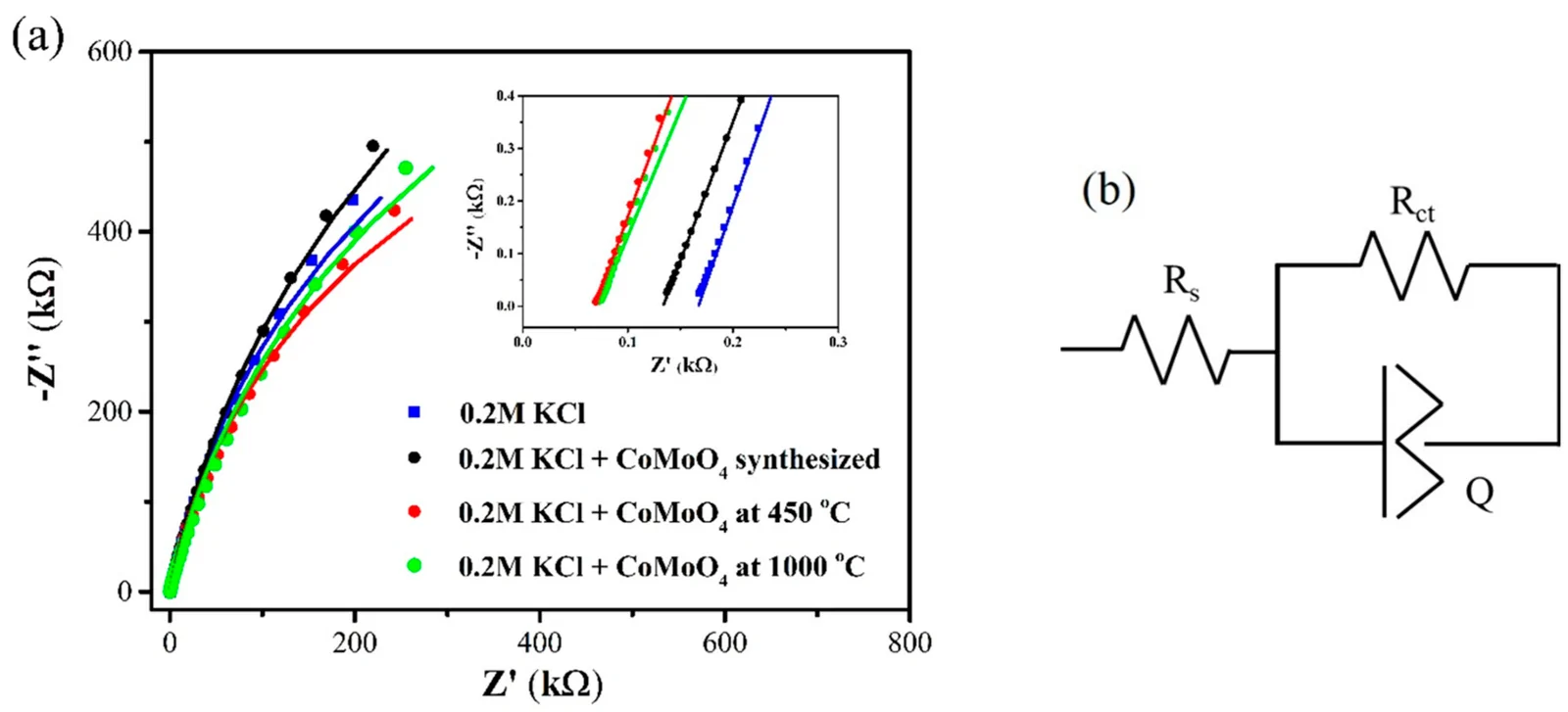
(**a**) Nyquist plots recorded in 0.2 mol dm^−3^ KCl with and without the added CoMoO_4_ samples (inset: high-frequency region) and (**b**) the modified Randles equivalent circuit used to fit the spectra.

The Nyquist plots recorded in 0.2 mol dm^−3^ KCl, with and without the added samples, are presented in Figure 9a. The spectra consist of incomplete semicircles, indicating a predominantly capacitive behaviour of the electrode interface. Since the measurements were performed at open-circuit potential in an electrolyte free of electroactive species, high charge-transfer resistances are expected, the only anticipated faradaic process being oxide formation at the Pt surface. The spectra were fitted with a modified Randles equivalent circuit (Figure 9b) comprising the solution resistance (*R*_s_) in series with the parallel combination of a constant-phase element (*Q*), representing the double-layer capacitance, and the charge-transfer resistance (*R_ct_*).

The constant-phase element (CPE) accounts for the non-ideal capacitive response; its impedance is given by [26]:

(5) *Z* = 1/*Y* = 1/[(*i ω*)*^n^* Q]

where *Y* is the CPE admittance, i is the imaginary unit, *ω* is the angular frequency, *n* is the CPE exponent (associated with surface inhomogeneity), and *Q* is the magnitude of the admittance at *ω* = 1 rad s^−1^. Depending on the value of *n*, the CPE reduces to a pure capacitance (*n* = 1), a pure resistance (*n* = 0), or a Warburg impedance (*n* = 0.5).

The effective double-layer capacitance was calculated from the CPE parameters using the Brug relation [26]. Because *R_ct_* >> *R_s_* for all systems studied here (MΩ vs. hundreds of Ω), the simplified form applies:

(6) *C_eff_* = *Y*_0_^1*/n*^* · R_s_*^(1*−n)/n*^

The high-frequency intercept on the real axis corresponds to the combined series resistance *R_s_*, which comprises the intrinsic resistance of the electrode material, the ionic resistance of the electrolyte, and the contact resistance. The addition of the CoMoO_4_ powders lowered *R_s_* in every case (from 193 Ω for the blank to 69–142 Ω) (Table 4). Because even complete dissolution of the added powder would change the ionic strength of 0.2 mol dm^−3^ KCl by less than 2%, this decrease cannot arise from increased electrolyte conductivity; it more likely reflects particles in contact with, or deposited on, the Pt surface and the associated change in the effective current path. The charge-transfer resistance, by contrast, did not follow a single trend: *R_ct_* decreased only for CoMoO_4_ at 450 °C (1.29 vs. 1.60 MΩ for the blank), remained essentially unchanged for CoMoO_4_ at 1000 °C (1.63 MΩ), and increased for CoMoO_4_ as-synthesized (2.09 MΩ) (Table 4). The lower *R_ct_* of CoMoO_4_ at 450 °C suggests that species released from the powder, or the solid phase itself, facilitate charge transfer, possibly acting as redox mediators at the Pt/electrolyte interface. The increase observed for CoMoO_4_ as-synthesized is consistent with partial blocking of the Pt surface by adsorbed or leached species and residual amorphous/hydroxylated surface phases—formed during the as-synthesized stage of incomplete crystallization—that lack the well-defined mediating sites present in the more crystalline powders.

**Table 4:** Impedance parameters extracted from the Nyquist plots and the corresponding effective capacitances, *C*_eff_, derived via the Brug model [26].

	*R_s_* (Ω)	*R_ct_* (MΩ)	*Y*1 (µS s^n1^)	*n*1	*C_eff_* (µF)	*χ* ^2^
0.2 M KCl	193	1.60	2.76	0.9	1.194	0.14
0.2 M KCl + CoMoO_4_ as-synthesized	142	2.09	2.54	0.9	1.053	0.04
0.2 M KCl + CoMoO_4_ at 450 °C	69	1.29	2.65	0.9	1.018	0.17
0.2 M KCl + CoMoO_4_ at 1000 °C	72	1.63	1.95	0.9	0.728	0.11

The effective double-layer capacitance, *C_eff_*, decreased systematically from 1.19 µF for the blank to 0.73 µF for the 1000 °C sample. This attenuation is attributed to the non-faradaic adsorption of leached species or fine particulate matter onto the Pt surface: displacement of compact water molecules within the Helmholtz layer by bulkier adsorbed species increases the effective double-layer thickness and lowers the local dielectric permittivity, thereby reducing the capacitance. Overall, CoMoO_4_ at 450 °C exhibits the lowest R_ct_ and one of the two lowest R_s_ values, indicating the most pronounced modification of the Pt/electrolyte interface (Table 4). Because these measurements were performed in KCl, in the absence of H_2_O_2_, the EIS parameters describe interfacial properties and not peroxidase-mimetic activity; their relationship to the BR and CV results is correlative rather than mechanistic.

### 3.5. Descriptor Φ = ρ_α_ × S_BET_: Scope and Limitations

The four independent datasets—FTIR, BR, CV and EIS—show that macroscopic kinetic reactivity in this series of GNP-derived CoMoO_4_ powders is not determined by the bulk crystallographic phase alone. We examine here to what extent it can be rationalized by the empirical, semi-quantitative descriptor *Φ* = *ρ_α_* × *S_BET_*, where *ρ_α_* is the *α*-type FTIR band-area fraction (Section 3.2) and *S_BET_* is the BET specific surface area. Three observations are relevant:

(1) The 450 °C powder (bulk-*β* by XRD) is the most active sample in the BR test and the only one that changes the log I_p_–log *ν* slope in CV. It suppresses BR oscillations at ≤0.002 g, shows the lowest R_ct_ in EIS, and gives CE kinetics in CV. Although its *S_BET_* exceeds the as-synthesized sample by only 9%, FTIR deconvolution (Table 3) gives *ρ_α_* = 0.90 ± 0.07 for the 450 °C powder—the highest value in the series—indicating the largest relative contribution of octahedral-type Mo–O environments. The corresponding *Φ* = 21.5 m^2^ g^−1^ is 2.1-fold larger than for the as-synthesized powder (*Φ* = 10.3 m^2^ g^−1^) and 1.9-fold larger than for the 1000 °C sample (*Φ* = 11.4 m^2^ g^−1^).

(2) The 1000 °C powder (bulk-*α*) is the least active per gram. Its fully octahedral Mo^6+^ coordination is expected to confer the highest per-site Lewis acidity [8,9], but calcination reduces *S_BET_* to 15 m^2^ g^−1^ and strongly reduces the porosity, lowering *Φ* to a value comparable to that of the as-synthesized powder. This is the apparent paradox: the bulk phase expected to be the most Lewis-acidic is the least reactive.

(3) The as-synthesized powder lies between the two in activity: moderate S_BET_, the lowest α-type band-area fraction, and a *Φ* value indistinguishable within uncertainty from that of the 1000 °C powder. Its BR slope (5172 s g^−1^) is four times the 1000 °C value, and its suppression threshold is at least 20-fold higher than that of the 450 °C sample, while Φ reproduces the second difference but not the first (see below).

It should be noted that *Φ* does not reproduce the BR activity order of the as-synthesized and 1000 °C powders: the as-synthesized powder is about four times more active per gram (BR slopes 5172 ± 406 vs. 1278 ± 80 s g^−1^; Section 3.3.3), whereas their *Φ* values (10.3 ± 5.5 and 11.4 ± 0.3 m^2^ g^−1^) are indistinguishable within uncertainty. Even after normalization of the BR slopes to S_BET_, the as-synthesized powder remains about 2.8 times more active (≈235 vs. ≈85 s m^−2^). Part of this discrepancy reflects a known limitation of BET surface area as a measure of the area accessible to substrates in solution: grains of the 1000 °C sample, with their strongly reduced porosity (Figure 1c), present mainly their external surface to aqueous H_2_O_2_, so the effectively accessible area is likely smaller than the BET value, whereas the porous as-synthesized morphology allows diffusive access through its open pore network. In addition, factors not included in *Φ*—Co^2+^/Co^3+^ redox chemistry, oxygen vacancies, surface hydroxylation (including the residual amorphous and hydroxylated phases of the as-synthesized powder), and defect density—are likely to contribute. The descriptor *Φ* therefore provides a partial rationalization of the observed reactivity trend by combining structural (*ρ_α_*) and textural (*S_BET_*) contributions. However, it does not reproduce the activity order of the as-synthesized and 1000 °C samples, indicating that additional factors, such as phase composition, crystallite size, defect concentration, or the nature of surface Co and Mo species, also influence the reactivity. Validation of *Φ* on a broader set of samples would be required to establish its predictive value.

Together with the photocatalytic results reported previously for the same powders [6], in which the 1000 °C powder was the most efficient photocatalyst, the present data suggest that calcination temperature may affect bulk-controlled (photocatalytic) and surface- and texture-controlled (peroxidase-mimetic) behaviour in different ways. Because this comparison combines data from two separate studies, it should be regarded as a hypothesis for future work rather than as a result of the present study.

The descriptor Φ is currently defined semi-quantitatively because ρ_α_ is estimated from relative FTIR band intensities rather than by a direct surface-site titration. Accordingly, Φ should presently be regarded as a comparative descriptor for the investigated sample series rather than as an absolute measure of Lewis acid site density. Placing *Φ* on a rigorous quantitative footing—through, for example, X-ray photoelectron spectroscopy of the Co 2p, Mo 3d and O 1s levels (Co^3+^/Co^2+^ ratio, oxygen vacancies), electron paramagnetic resonance for vacancy-related defects, temperature-programmed reduction of surface Mo^6+^, or infrared spectroscopy of adsorbed pyridine or CO (Lewis and Brønsted acidity)—remains an open goal and would allow the contributions of the individual factors to be separated.

## 4. Conclusions

Three glycine–nitrate-derived CoMoO_4_ nanopowders—as-synthesized and calcined at 450 °C (both *β*-CoMoO_4_ by XRD) and calcined at 1000 °C (*α*-CoMoO_4_)—were compared by ATR-FTIR spectroscopy, the Briggs–Rauscher reaction, cyclic voltammetry and electrochemical impedance spectroscopy. The following conclusions apply to this three-sample series.

(1) Deconvolution of the Mo–O stretching region with a uniform Voigt line shape resolves octahedral (*α*-type) and tetrahedral (*β*-type) Mo^6+^ components in both *β*-classified powders, indicating short-range coordination heterogeneity not detected by powder XRD. The band-area fraction ρ_α_ is a semi-quantitative, relative quantity and not a direct measure of Lewis acid site density.

(2) The 450 °C powder is the most active sample: it suppresses BR oscillations at ≤0.002 g (threshold 0.0011–0.0020 g), whereas the as-synthesized powder still oscillates at 0.040 g (slope 5172 ± 406 s g^−1^, n = 3) and the bulk-*α* 1000 °C powder is about four times less active per gram than the as-synthesized powder. Complete H_2_O_2_ depletion was not demonstrated, and a contribution of dissolved Co/Mo species to the oscillatory response cannot be completely excluded.

(3) CV in H_2_O_2_-containing electrolyte supports the highest activity of the 450 °C powder; EIS in KCl provides correlative evidence of interfacial modification.

(4) The descriptor *Φ* = *ρ_α_* × *S_BET_* identifies the most active powder but does not discriminate between the as-synthesized and 1000 °C powders. Co^2+^/Co^3+^ redox chemistry, oxygen vacancies, surface hydroxylation, and defects are likely additional contributors.

(5) Within this series, bulk phase identity alone does not predict peroxidase-mimetic activity. Direct surface-acidity measurements (pyridine-FTIR, XPS) and a larger set of samples are needed to test whether the proposed relationship between local coordination, accessibility and activity holds more generally.

## Figures and Tables

**Table 1:** Bulk structural and textural parameters (XRD, DTA, BET from ref. [6]). *D*, Scherrer crystallite size; *S_BET_*, BET surface area; *E_g_*, optical band gap (Kubelka–Munk).

Sample	Phase (XRD)	Mo^6+^ Coord.	V_uc_ (Å^3^)	D (nm)	*S_BET_* (m^2^ g^−1^)	E_g_ (eV)	Morphology
CoMoO_4_ as-synthesized	*β*-CoMoO_4_	Tetrahedral (MoO_4_)	703.1	6.6	22	1.8	Sponge-type, highly porous network
CoMoO_4_ at 450 °C	*β*-CoMoO_4_	Tetrahedral (MoO_4_)	700.8	5.2	24	2.8	Grape-like agglomerates of fine crystallites
CoMoO_4_ at 1000 °C	*α*-CoMoO_4_	Octahedral (MoO_6_)	600.7	38.4	15	2.3	Faceted polyhedral grains

**Table 2:** FTIR band positions from Voigt deconvolution of the as-synthesized, 450 °C and 1000 °C CoMoO_4_ spectra, with their assignments. Assignments follow Rahulan et al. [24], Cherian et al. [25], Costa et al. [12], Rodriguez et al. [13] and Lakhlifi et al. [14]; bond-length considerations follow Hardcastle and Wachs [27].

ν (cm^−1^)	As-Synth.	450 °C	1000 °C	Vibrational Mode	Local Coordination/Structural Origin	Phase Diagnostic	Ref.
≈968–937	968, 948	964	937	Terminal Mo=O stretch (higher-wavenumber component)	Isolated MoO_4_ tetrahedra (*β*-type)	*β*-type	[14,24,25,27]
≈926–899	925	926	899	Mo–O stretch, distorted octahedra	Distorted MoO_6_ (Mo_4_O_16_ tetramers; *α*-type)	*α*-type	[12,13,14,24]
≈841	841	841	— *	Asymmetric Mo–O–Mo bridge stretch	Corner-/edge-shared bridging O	Both	[24,25]
≈780–670	780, 719, 693	778, 691	670	Symmetric Mo–O–Mo/chain-framework modes	Bridging backbone of monoclinic lattice; ≈690–700 cm^−1^ *α*-sensitive	Both	[24,25]
≈657–624	657	652, 624	—	Co–O–Mo inter-polyhedral coupling	Shared-O linkages between Co and Mo sublattices	Both	[24]
≈588–560	560	568	588	CoO_6_ bending + lattice phonons	Octahedral Co–O (common to both polymorphs)	Both	[24,25]

* The 1000 °C spectrum was fitted with only four components; the broad 899 cm^−1^ band probably also contains the Mo–O–Mo bridge contribution.

**Table 3:** Voigt-profile deconvolution of the Mo–O stretching region (≈870–1000 cm^−1^): band positions of the *α*-type and *β*-type components (*ν_α_*, *ν_β_*), analytic band areas A(*α*) and A(*β*) (a.u.), relative *α*-type band-area fraction *ρ_α_* = A(*α*)/[A(*α*) + A(*β*)] (± uncertainty propagated from the fitted-area standard errors), BET surface area *S_BET_* [6], comparative descriptor *Φ* = *ρ_α_ × S_BET_*, and coefficient of determination of the fit R^2^. All spectra were fitted with the same Voigt line shape.

Sample	*ν_α_* (cm^−1^)	*A*(*α*) (a.u.)	*ν_β_* (cm^−1^)	*A*(*β*) (a.u.)	*ρ* *_α_*	*S_BET_* (m^2^ g^−1^)	*Φ* = *ρ_α_* × *S_BET_* (m^2^ g^−1^)	*R* ^2^
As-synthesized	925.4	2.317	956.7 ^a^	2.623 ^a^	0.47 ± 0.25	22	10.3 ± 5.5	0.9981
450 °C	925.9	11.041	964.4	1.271	0.90 ± 0.07	24	21.5 ± 1.7	0.9990
1000 °C	898.6	23.367	937.3	7.282	0.76 ± 0.02	15	11.4 ± 0.3	0.9925

^a^ Sum of the components at 948.0 cm^−1^ (area 1.489) and 968.2 cm^−1^ (area 1.134); *ν_β_* is their area-weighted centre.

## Data Availability

The original contributions presented in this study are included in the article/Supplementary Material. Further inquiries can be directed to the corresponding author.

## Supplementary Materials

The following supporting information can be downloaded at https://www.mdpi.com/article/10.3390/ma19204318/s1, Figure S1: Cyclic voltammograms recorded in the (a) MA–HClO_4_; (b) MnSO_4_–HClO_4_; (c) KIO_3_–HClO_4_, and (d) H_2_O_2_–HClO_4_ electrolytes without (dashed) and with (solid) the added CoMoO_4_ at 450 °C.; Figure S2: Cyclic voltammograms recorded in the (a) MA–HClO_4_; (b) MnSO_4_–HClO_4_; (c) KIO_3_–HClO_4_, and (d) H_2_O_2_–HClO_4_ electrolytes without (dashed) and with (solid) the added CoMoO_4_ at 1000 °C.

## References

[1] Mai L.Q., Yang F., Zhao Y.L., Xu X., Xu L., Luo Y.Z. (2011). Hierarchical MnMoO_4_/CoMoO_4_ heterostructured nanowires with enhanced supercapacitor performance. Nat. Commun..

[2] Veerasubramani G.K., Krishnamoorthy K., Radhakrishnan S., Kim N.-J., Kim S.J. (2014). Synthesis, characterization, and electrochemical properties of CoMoO_4_ nanostructures. Int. J. Hydrogen Energy.

[3] Xu Z., Li Z., Tan X., Holt C.M.B., Zhang L., Amirkhiz B.S., Mitlin D. (2012). Supercapacitive carbon nanotube-cobalt molybdate nanocomposites prepared via solvent-free microwave synthesis. RSC Adv..

[4] Zhang Y.J., Rodríguez-Ramos I., Guerrero-Ruiz A. (2000). Oxidative dehydrogenation of isobutane over magnesium molybdate catalysts. Catal. Today.

[5] Kojima R., Aika K.-I. (2001). Cobalt molybdenum bimetallic nitride catalysts for ammonia synthesis. Appl. Catal. A Gen..

[6] Rosić M., Zarubica A., Šaponjić A., Babić B., Zagorac J., Jordanov D., Matović B. (2018). Structural and photocatalytic examination of CoMoO_4_ nanopowders synthesized by GNP method. Mater. Res. Bull..

[7] Choi G.-K., Kim J.-R., Yoon S.H., Hong K.S. (2007). Microwave dielectric properties of scheelite (A = Ca, Sr, Ba) and wolframite (A = Mg, Zn, Mn) AMoO_4_ compounds. J. Eur. Ceram. Soc..

[8] Wang Z., Ju P., Zhang Y., Jiang F., Ding H., Sun C. (2020). CoMoO_4_ nanobelts as efficient peroxidase mimics for the colorimetric determination of H_2_O_2_. Microchim. Acta.

[9] Busca G. (1999). The surface acidity of solid oxides and its characterization by IR spectroscopic methods. An attempt at systematization. Phys. Chem. Chem. Phys..

[10] Wachs I.E. (2005). Recent conceptual advances in the catalysis science of mixed metal oxide catalytic materials. Catal. Today.

[11] Robertson L.C., Gaudon M., Jobic S., Deniard P., Demourgues A. (2011). Investigation of the First-Order Phase Transition in the Co_1−_*_x_*Mg*_x_*MoO_4_Solid Solution and Discussion of the Associated Thermochromic Behavior. Inorg. Chem..

[12] Costa R.K.S., Teles S.C., Siqueira K.P.F. (2021). The relationship between crystal structures and thermochromism in CoMoO_4_. Chem. Pap..

[13] Rodriguez J.A., Chaturvedi S., Hanson J.C., Albornoz A., Brito J.L.J. (1998). Electronic Properties and Phase Transformations in CoMoO_4_ and NiMoO_4_: XANES and Time-Resolved Synchrotron XRD Studies. Phys. Chem. B.

[14] Lakhlifi H., El Jabbar Y., Benchikhi M., Er-Rakho L., Durand B., El Ouatib R. (2022). Nanocrystalline transition metal (CoMoO_4_) prepared by sol gel method: Correlation between powder colors and α/β phase transformations. Inorg. Chem. Commun..

[15] Cai G., Chin Y.-H.C. (2025). Thermochemical and Kinetic Correlations of Redox and Lewis Sites on Cobalt–Molybdenum Oxides: Illustrated with Alkanol-O_2_ Catalysis. ACS Catal..

[16] Zagorac D., Schön J.C., Rosić M., Zagorac J., Jordanov D., Luković J., Matović B. (2017). Theoretical and Experimental Study of Structural Phases in CoMoO_4_. Cryst. Res. Technol..

[17] Rosić M., Milošević M., Čebela M., Dodevski V., Lojpur V., Čakar U., Stopic S. (2025). Spectroscopic and Morphological Examination of Co_0.9_R_0.1_MoO_4_ (R = Ho, Yb, Gd) Obtained by Glycine Nitrate Procedure. Materials.

[18] De Kepper P., Epstein I.R. (1982). Mechanistic study of oscillations and bistability in the Briggs-Rauscher reaction. J. Am. Chem. Soc..

[19] Briggs T.S., Rauscher W.C. (1973). An oscillating iodine clock. J. Chem. Educ..

[20] Furrow S.D., Noyes R.M. (1982). The oscillatory Briggs-Rauscher reaction. 1. Examination of subsystems. J. Am. Chem. Soc..

[21] Cervellati R., Höner K., Furrow S.D., Neddens C., Costa S. (2001). The Briggs-Rauscher reaction as a test to measure the activity of antioxidants. Helv. Chim. Acta.

[22] Pagnacco M.C., Maksimović J.P., Mudrinić T.M., Mojović Z.D., Nedić Z.P. (2019). Briggs-Rauscher reaction as a novel electrochemical detector for phosphate tungsten and phosphate molybdenum bronzes. J. Electroanal. Chem..

[23] Bošković S., Matović B., Vlajić M., Krstić V. (2007). Modified glycine nitrate procedure (MGNP) for the synthesis of SOFC nanopowders. Ceram. Int..

[24] Rahulan K.M., Flower N.A.L., Sujatha R.A., Mathiyarasu V. (2018). Nonlinear optical absorption studies of CoMoO_4_ hybrid nanostructures. J. Mater. Sci. Mater. Electron..

[25] Cherian C.T., Reddy M.V., Haur S.C., Chowdari B.V.R. (2013). Interconnected network of CoMoO_4_ submicrometer particles as high capacity anode material for lithium ion batteries. ACS Appl. Mater. Interfaces.

[26] Brug G.J., van den Eeden A.L.G., Sluyters-Rehbach M., Sluyters J.H. (1984). The analysis of electrode impedances complicated by the presence of a constant phase element. J. Electroanal. Chem..

[27] Hardcastle F.D., Wachs I.E. (1990). Determination of molybdenum–oxygen bond distances and bond orders by Raman spectroscopy. J. Raman Spectrosc..

[28] Pagnacco M., Maksimović J., Mudrinić T., Banković P., Nedić-Vasiljević B., Milutinović-Nikolić A. (2020). Oscillatory Briggs-Rauscher Reaction as “Fingerprint” for Bentonite Identification: The Fine-Tuning of Oscillatory Dynamics with Addition of Clay. ChemistrySelect.

[29] Ray S.K., Bastakoti B.P. (2024). Improved supercapacitor and oxygen evolution reaction performances of morphology-controlled cobalt molybdate. Int. J. Hydrogen Energy.

[30] Bard A.J., Faulkner L.R. (2001). Electrochemical Methods: Fundamentals and Applications.

[31] Orazem M.E., Tribollet B. (2017). Electrochemical Impedance Spectroscopy.

