# Supplementary material for: Local Mo6+ Coordination and Textural Accessibility, Rather than Bulk Phase, Correlate with the Peroxidase-Mimetic Reactivity of CoMoO4 Nanopowders
Source: Materials (Basel). 2026 Oct 14;19(20):4318. doi: 10.3390/ma19204318 (PMC13707084; doi:10.3390/ma19204318)
Supplement: Supplementary file 1 [file materials-19-04318-s001.zip › materials-4555114-supplementary.pdf]

Local Mo^{6+} Coordination and Textural Accessibility, Rather than Bulk Phase, Correlate with the Peroxidase-Mimetic Reactivity of $CoMoO_4$ Nanopowders

Milena Rosić [1], Zorica Mojović [2], Maja C. Pagnacco [2], Jelena V. Senćanski [3], Jelena P. Maksimović [4], Dragana Jordanov [1] and Maria Čebela [1,*]

[1] "Vinča" Institute of Nuclear Sciences, National Institute of the Republic of Serbia, University of Belgrade, Mike Petrovića Alasa 12-14, 11351 Belgrade, Serbia; mrosic@vin.bg.ac.rs (M.R.); djordano@vin.bg.ac.rs (D.J.)
[2] Institute of Chemistry, Technology and Metallurgy, University of Belgrade, Njegoševa 12, 11000 Belgrade, Serbia; zorica.mojovic@ihtm.bg.ac.rs (Z.M.); maja.pagnacco@ihtm.bg.ac.rs (M.C.P.)
[3] Institute for Multidisciplinary Research, University of Belgrade, Kneza Višeslava 1, 11030 Belgrade, Serbia; jelenasencanski@gmail.com
[4] Faculty of Physical Chemistry, University of Belgrade, Studentski trg 12-16, 11158 Belgrade, Serbia; jelena.maksimovic@ffh.bg.ac.rs
* Correspondence: mcebela@vin.bg.ac.rs

Supporting Information

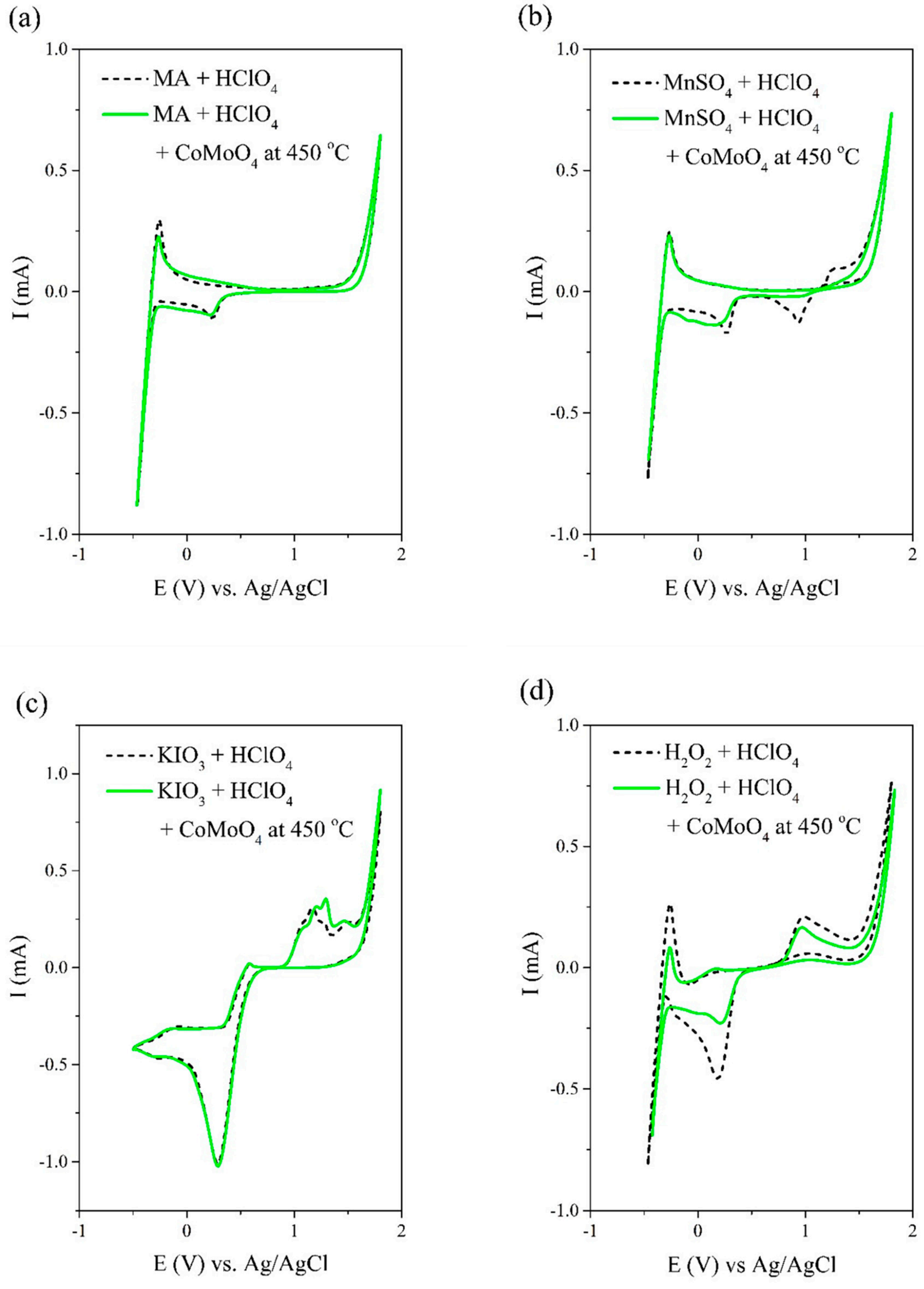

Figure S1. Cyclic voltammograms recorded in the a) MA–$HClO_4$; b) $MnSO_4$–$HClO_4$; c) KIO_3–$HClO_4$, and d) H_2O_2–$HClO_4$ electrolytes without (dashed) and with (solid) the added $CoMoO_4$ at 450 °C.

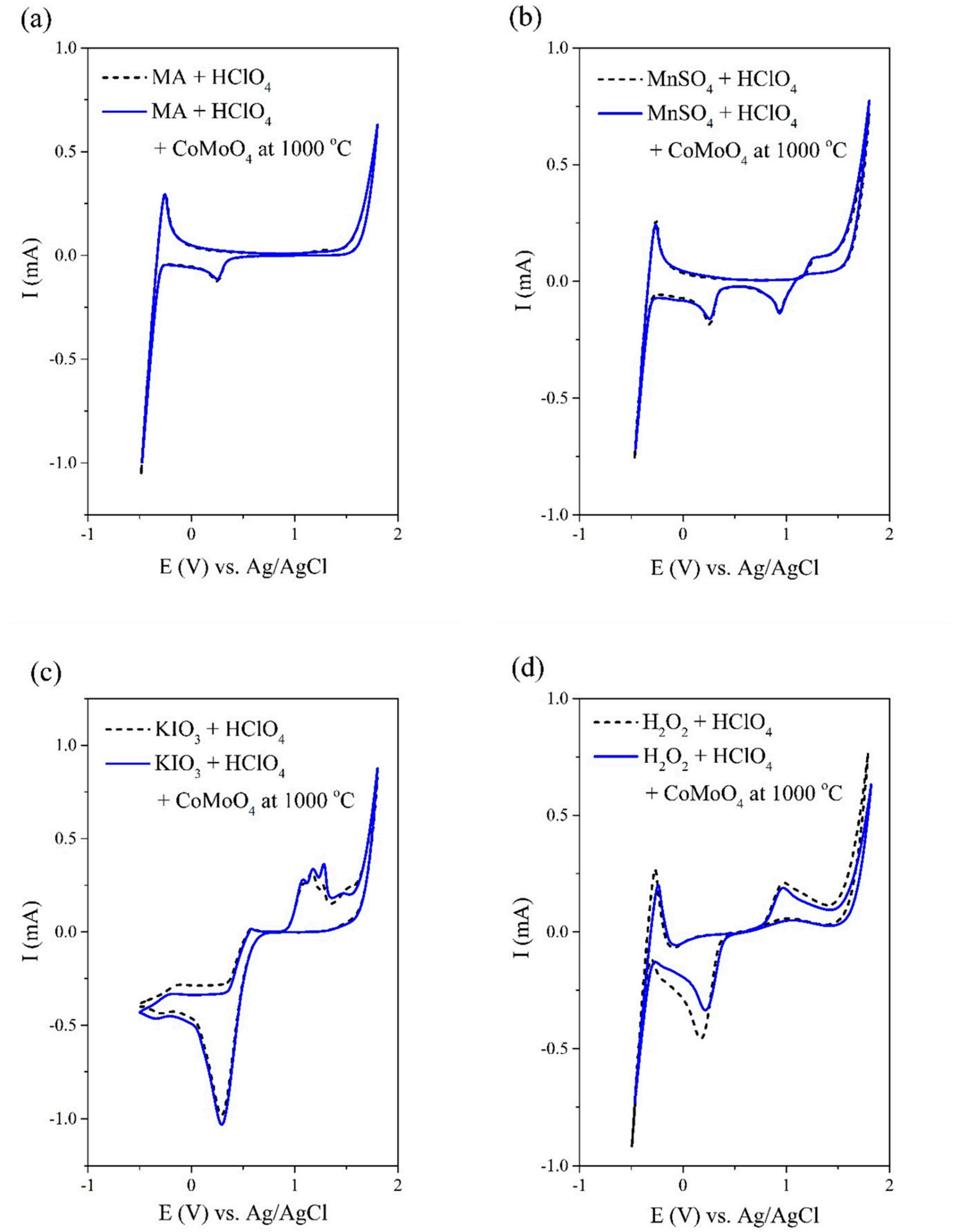

Figure S2. Cyclic voltammograms recorded in the a) MA–$HClO_4$; b) $MnSO_4$–$HClO_4$; c) KIO_3–$HClO_4$; and d) H_2O_2–$HClO_4$ electrolytes without (dashed) and with (solid) the added $CoMoO_4$ at 1000 °C.
